# Human–AI co-research on design and evaluation of Embodied Conversational Agent in rehabilitation contexts

**DOI:** 10.3389/frobt.2026.1758391

**Published:** 2026-03-26

**Authors:** Anna Lekova, Paulina Tsvetkova, Tsvetelin Stefanov

**Affiliations:** 1 Bulgarian Academy of Sciences, Institute of Robotics, Sofia, Bulgaria; 2 Centre of Competence: Intelligent Mechatronic, Eco- and Energy-Saving Systems and Technologies, Lab C5.3 Robotics and Mechatronics, Sofia, Bulgaria; 3 University of Library Studies and Information Technologies, Faculty of Information Science, Sofia, Bulgaria

**Keywords:** Design-Based Research, Embodied Conversational Agents, Furhat robot, human–AI co-design, LLMs, prompt engineering, synthetic data generation

## Abstract

**Introduction:**

Despite strong evidence that repetitive home-based rehabilitation improves functional recovery after stroke, current delivery models still show gaps in continuity of care and patient engagement. AI-driven Embodied Conversational Agents (ECAs) could provide personalized home support through natural-language guidance on prescribed exercises, reinforcement of neuroplasticity, clarification of therapeutic principles, and motivational support. However, clinical deployment remains challenging. Many robotic platforms lack real-time interaction capabilities such as speech processing, gesture execution, and attention tracking, while Large Language Models (LLMs) may produce factual errors or inconsistent responses. Early development is also constrained by limited access to real users due to practical and ethical considerations.

**Methods:**

To address these challenges, we propose a Design-Based Research methodology for human–AI co-design and evaluation of ECAs (co-AI DBR), where generative AI facilitates iterative cycles of design, testing, and refinement. Co-AI DBR combines synthetic patient generation with real-code execution to simulate, emulate, and evaluate the ECA platform and its LLM-based conversational pipeline. To validate the method in a post-stroke rehabilitation context, a virtual ECA was first tested with synthetic patients to assess technical implementation and accuracy of LLM responses. A pilot deployment using the Furhat robot as an ECA was then conducted with patient relatives and rehabilitation professionals to evaluate the voice interface and augmented communication.

**Results:**

LLM responses to questions from real participants showed higher lexical diversity (MTLD ≈ 134 vs. 93.9) and lower repetition (Yule’s K ≈ 66.8 vs. 115.4) than responses to synthetically generated questions. Responses remained factually consistent, with no contradictions and complete gender invariance, although slightly lower hapax rates were observed (88.8% vs. 99.4%). Usability scores were higher among relatives (M = 86.67) than professionals (M = 72.50), while Intrinsic Motivation Inventory scores indicated similarly high motivation in both groups (M = 6.32 vs. 6.12).

**Discussion:**

The results suggest that co-AI DBR can support early design and evaluation of ECAs when direct patient testing is limited. By combining synthetic patient generation with real-code execution, generative AI supports iterative knowledge building during the prototyping and refinement of LLM-based ECAs. This methodology enables the practical development of ECA to support home-based post-stroke rehabilitation.

## 1 Introduction

Despite substantial evidence supporting adequate and repetitive home-based rehabilitation to optimize functional recovery and independence in daily living, current rehabilitation delivery models continue to exhibit critical gaps and limitations. As a result, many patients, such as those recovering from stroke, experience persistent barriers, including limited access to therapists, insufficient therapy, low engagement in self-management exercises and reduced motivation. Empirical research consistently highlights the importance of adequate therapeutic dosage through task-specific and repetitive practice at home to support experience-dependent neuroplasticity and long-term functional recovery (Lang et al., 2007; Winstein et al., 2016; Fu et al., 2025). According to stroke guidelines in (Winstein et al., 2016), rehabilitation programs over 3 hours per day, produce significantly greater functional improvements, yet real-world daily activity levels remain low and do not align with evidence-based recommendations. Furthermore, while stroke rehabilitation has traditionally focused on physical recovery, emerging evidence highlights the need to address long-term psychosocial, as well as speech and language, impairments. Recent studies emphasize that home-based rehabilitation should integrate psychosocial wellbeing into stroke recovery practices (Fu et al., 2025), with broader self-management approaches shown to enhance long-term outcomes (Richards et al., 2023; Lynch et al., 2025). To address these gaps, we propose an embodied, conversational AI-driven personal assistant, commonly referred to as embodied conversational agent (ECA), to enhance continuity of care at home. It is designed to complement, not replace, therapists by providing personalized, natural-language support that reinforces experience-dependent neuroplasticity, reminds patient of evidence-based home-exercise guidance, explains therapeutic principles when needed, and offers emotional and motivational support to sustain engagement, all without making any clinical decisions.

Conversational AI, either assisted or driven by Large Language Models (LLMs), has become a core component in the advancement of ECAs to support intuitive and context-aware natural language interactions. ECAs may appear as virtual avatars or physical robots, which are often categorized as Socially Assistive Robots (SARs). Recent surveys highlight the potential of SARs across various domains, particularly in rehabilitation and special education (Carnevale et al., 2025; He et al., 2025; Hung et al., 2025; Evdokia et al., 2025). LLM-enhanced SARs (Nicora et al., 2025; Pinto-Bernal et al., 2025; Evdokia et al., 2025) have demonstrated increased motivation and engagement and have supported self-management interventions across cognitive, physical and psychological rehabilitation. However, integrating LLMs into physical embodied agents is challenging, because they are not originally designed for such deployment (Pinto-Bernal et al., 2025). Many SARs lack native support for real-time speech processing, gesture execution and interpretation, attention tracking and long-term memory management, all of which are required to operationalize LLM-driven interaction. System-level limitations of current LLMs, such as response latency, hallucinations, factual errors, inconsistency and limited reliability, further restrict their direct integration as ECAs, particularly in clinical and rehabilitation settings. To address these technical and practical limitations, research must combine engineering solutions with systematic assessments of the clinical safety of LLM-powered ECA interactions.

One of the earliest conversational SARs studied in therapeutic and rehabilitation settings was THERAPIST (Calderita et al., 2014). In subsequent years, advances in sensing, dialogue management and affective computing facilitated dynamic dialogue generation and adaptive coaching strategies, allowing ECAs to more effectively respond to users’ cognitive, emotional and behavioral states in both physical and psychological therapy (He et al., 2025; Hung et al., 2025; Nicora et al., 2025). Early empirical rehabilitation studies (Feingold-Polak and Levy-Tzedek, 2021; Langer and Levy-Tzedek, 2021) employed rule-based or classical dialogue systems to tailor interactions to users’ cognitive and emotional needs. In the iTakeCharge project (Richards et al., 2023), the evidence-based, narrative-style Take Charge intervention (Fu et al., 2020) was applied and has been shown to improve post-stroke disability, self-efficacy, and quality of life.

User feedback is critical for the design and evaluation of ECAs tailored to individual rehabilitation, as it supports their relevance, usability and acceptance (Sonlu et al., 2025; Feingold-Polak and Levy-Tzedek, 2021). However, during the early stages of design and evaluation the access to diverse real users is often limited due to practical or logistical constraints and involving vulnerable populations may be ethically inappropriate. Authors in Kocaballi et al. (2022) specifically identify in their section with limitations that recruiting vulnerable populations is difficult due to limited resources and community connections, and that most testing occurs in laboratories rather than real-world settings. The authors in Lee et al. (2025) also emphasize that empirical studies of conversational agents are frequently constrained by limit participant samples and challenges in engaging patients at early deployment stages. To overcome these limitations, we conducted simulations and emulations using synthetic interactions for early testing and validation of ECAs with generative AI (GenAI). GenAI can not only accelerate the design and testing of software solutions but also expand the design space prior to human deployment. Therefore, we propose the use of GenAI-generated Synthetic Digital Twins (SDTs) to emulate the operational pipeline of LLM-driven ECA through both frontend interactions and backend endpoints, enabling systematic knowledge extraction from these evaluations.

SDTs are AI-generated virtual patient representations designed to be contextually realistic, and clinically and ethically grounded. SDTs contain personal and clinical profiles, rehab goals, tracking clinical progress over time and issue-centered patterns that describe symptoms, concerns and preferences in natural language. Leveraging these datasets, we introduce a co-AI Design-Based Research (co-AI DBR) methodology that integrates human expertise with GenAI to facilitate the iterative design and simulation-driven validation of ECAs. GenAI acts as a knowledge-producing partner in DBR, embedded within a structured, simulation- and emulation-driven pipeline, where synthetic patient generation and real-code execution co-construct insights, expand the design space and accelerate iterative design refinement under constraints on direct human testing. In this context, synthetic data generation plays a critical role in producing realistic patient simulations. Emerging approaches that synthesize domain-specific datasets in data-scarce domains have demonstrated relevance and factual consistency. For example, DoPAMine (Arannil et al., 2024) uses seed prompts to guide the mining of domain-relevant examples for domain-specific pretraining and fine-tuning, whereas Precision at Scale (Rodríguez-de-Vera et al., 2024) enables scalable dataset generation through modular pretrained components. Leveraging LLMs’ ability to generate human-like responses with robust common-sense reasoning under few-shot learning (Brown et al., 2020), we adopt the domain-specific synthetic data generation strategy like in Arannil et al. (2024). This approach uses empirically validated prompts and follows methodology described by Syahputri et al. (2025), outlining prompt engineering as a structured and empirically tested design activity that ensures semantic diversity, factual consistency and domain-grounded outputs.

We used quantitative measures to evaluate the realism of SDTs and the semantic diversity, factual consistency and clinical grounding of ECA responses produced by LLMs. These assessments included response latency, lexical diversity, hapax legomena rate, self-contradictory hallucinations, user-model contradictions, factual inconsistencies and bias. In addition, domain specialists conducted qualitative reviews of the ECA generated responses to assess contextual realism, emotional appropriateness, and clinical relevance. After the pilot human deployment, in which the ECA received real questions, the LLM-generated responses were evaluated using the same procedure. We further assessed the voice-based interface and the realism of interactions with the Furhat robot as an ECA using questionnaires evaluating perceived ease of use, interaction quality, perceived usefulness and user engagement. Finally, we introduce a risk management approach based on the framework proposed by Iliuță et al. (2024), considering data security, model reliability, ethical compliance and human-in-the-loop evaluation to ensure clinical alignment and contextual realism. We refined the framework and incorporate two additional risks: addressing “hallucination” as a common AI-model accuracy risk, and assessing the ECA’s ability to avoid harmful or misleading responses while providing therapeutically appropriate and safe guidance for patients.

The objectives of this study are twofold: (1) to introduce a co-AI DBR methodology that integrates human expertise with generative AI capabilities to support the iterative design and assessment of ECAs using SDTs, which emulate real user interactions in a rehabilitation context; and (2) to validate this methodology in a post-stroke rehabilitation setting - first using SDTs interacting with a virtual ECA to assess the accuracy of LLM-generated responses and their ability to provide therapeutic explanations, emotional, and motivational support, and subsequently through a pilot human deployment with the Furhat robot, as a physical ECA instantiation, to evaluate the voice interface and augmented communication based on real patient concerns and their rehabilitation plans.

The primary research question guiding this study was: How can integrating human–AI co-research and emulative SDTs within a co-AI DBR framework advance iterative knowledge building for the development of ECAs that provide personalized, clinically aligned, and contextually realistic rehabilitation support, while enabling systematic evaluation of LLM-driven interactions and safe, ethically grounded deployment?

The paper is organized as follows: Section 2 presents the co-AI DBR methodology. Section 3 provides the validation approach in post-stroke rehabilitation settings and outcomes from each phase. Section 4 discusses the results, potential risks and future directions. Then, conclusions follow.

## 2 Methodology

**FIGURE 1:**
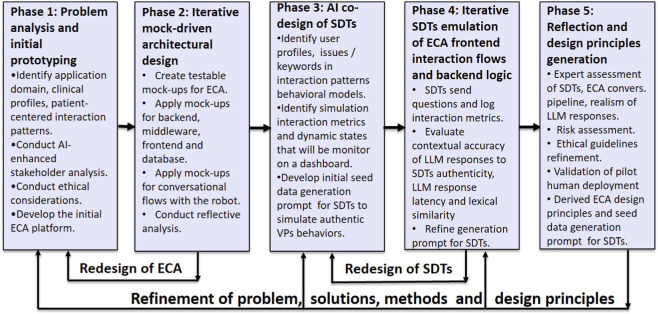
Five-Phase co-AI Design-Based Research framework integrating Synthetic Digital Twins (SDTs) for early-stage emulation of an Embodied Conversational Agent (ECA) in rehabilitation contexts.

This section presents the co-AI DBR methodology for the design and evaluation of embodied conversational agents in rehabilitation contexts (Figure 1). At the early stage of design, it uses synthetically generated digital twins and iterative prototype implementations to simulate the ECA architecture and its conversational pipeline. The SDTs emulate frontend interactions, backend processes and connections to real LLMs, allowing repeated testing across diverse patient behavioral patterns and psychological profiles. Generative AI is integrated into the early stages of the co-AI DBR framework through iterative, collaborative prompt development and refinement, where human designers and stakeholders jointly define prompts, constraints, and evaluation criteria to establish a clinically grounded reference for rehabilitation contexts. AI then generates SDTs that emulate realistic patient profiles and behaviors, which interact with the ECA using the same APIs and LLM endpoints as the real system, enabling end-to-end simulation of patient–coach interactions prior to deployment.

During Phase 1 of the co-AI DBR approach, the target application domain, featuring a patient-centered ECA as a rehabilitation coach, is identified, and user clinical profiles and interaction patterns are analyzed through combined AI-powered and developer- and stakeholder-directed analysis, adhering to preliminary ethical considerations. During the development of the initial ECA platform, GenAI can assist in programming backend endpoints and middleware, as well as in frontend and database design.

During Phase 2, a mock-driven architectural design approach is used to enable offline and cost-efficient iterative testing of the ECA software. GenAI can assist in creating initial testable mock-ups, which check function calls, instantiations, and return values, or inject controlled values during execution. Mocks are first used to test the backend endpoints and child processes and then applied to validate the interactions in the dialogue pipeline, enabling hundreds of tests to be executed, free from the constraints of human fatigue or behavioral variability. Then, mock-ups are used to assess conversational flow and speech patterns through simulated dialogues. By integrating the ECA with touch-enabled external display, rehabilitation can be supported by displaying files, images or short videos, exactly when they’re needed during speech.

During Phase 3, genAI is instructed to create SDTs using ground truth data that include static personal and clinical profiles, dynamic behavioral models (dialog prompts, task heuristics, dropout thresholds), live simulation metrics (tasks completed, errors, interaction time), and adaptive states (frustration, engagement, dropout probability). Through AI co-design across several iterations, seed data generation prompts are defined, and user profiles, interactions in dialogs, and dynamic states are identified for dashboard monitoring. Initial seed prompts generate realistic virtual patient behaviors, with each SDT exhibiting distinct traits, heuristics and possible limitations. Clinically realistic questions or concerns are featured, with expected responses containing at least ten keywords aligned with the SDT’s personal and clinical profiles, and dynamic states.

During Phase 4, SDTs emulate real user interaction behaviors with ECA operating as a rehabilitation coach and its associated functionalities over multiple sessions spanning a one-month period. The goal is to evaluate the realism, responsiveness, and therapeutic alignment of LLM-generated ECA responses while monitoring dynamic state updates for each SDT, reflecting gradual rehabilitation progress. Dynamic SDT states, such as mood and fatigue, are updated programmatically from the ECA software based on prior user utterances and simple heuristic models to change the SDT rehabilitation dynamics. Using puppeteer-based programmatic browser control, each SDT sends questions to the frontend and receives responses from the ECA, which are logged for real-time or offline analysis across personal, clinical, social, and linguistic dimensions. Online performance evaluation includes metrics such as LLM response time, task completion, and error rates based on adherence to expected keywords bound to each SDT issue-centered patterns, while LLM hallucination frequency and lexical characteristics are compared offline to patient profiles and authentic clinical documentation on the web. Based on these results, the ECA prompt, platform, and Phase 3 seed prompt may require iterative refinement to enrich SDT data with references to real-world rehabilitation guidelines and to change questions or concerns to be more grounded in evidence-based practice, thereby better reflecting SDT rehabilitation dynamics.

During Phase 5, experts evaluate the final iteration of the ECA platform and its conversational pipeline by analyzing logged interaction and performance metrics, assessing the realism of LLM responses, verifying the contextual relevance of SDTs and conducting risk assessments of ECA conversational pipeline for potential harm with unintended consequences. Subsequently, a pilot human deployment validates the platform’s real-world applicability and robustness by assessing performance, usability, and user acceptance of the ECA’s speech-based dialogue interface and embodiment, supporting real-time multimodal human–robot interaction in a realistic rehabilitation context. Based on ECA contextual relevance, LLM performance in ECA pipeline, and the validation of seed data generation prompt for SDTs, the design principles for ECAs in rehabilitation contexts are derived.

## 3 Materials and methods

This section validates the co–AI DBR methodology, highlighting its relevance in post-stroke rehabilitation by supporting user-centered design and self-directed rehabilitation outside clinical settings, contributing to more repetitive and holistic recovery. Implementation and validation aim to derive design principles for post-stroke rehabilitation ECAs, focusing on interaction quality, conversational consistency, seed-data prompts, SDTs usability and LLM responses, rather than clinical efficacy.

### 3.1 Materials

Furhat robot^1^, the next-generation humanoid robot with human-like conversational and social skills, serves as physical embodiment of conversational agent, enabling multimodal interaction through speech, facial expressions and gaze. This is essential in post-stroke context to promote engagement, social presence and long-term adherence, which are critical factors in home-based rehabilitation.

The system backend was implemented using a Node.js Express server and the python Furhat Realtime API. Automated testing employed Node.js Jest and Puppeteer. Speech synthesis and recognition were provided via Google Cloud and Microsoft Azure services. AI reasoning relied on remote cloud APIs, including bgGPT and NLPCloud. The evaluated LLMs for SDT generation were ChatGPT-5.0, Gemini-2.5-Pro, DeepSeek-V3.2 and Grok-3, while for LLM responses - LLaMA 3–70B, gpt-oss-120b and gemma-2-27b. All cloud services and LLMs are integrated in a modular and optional manner, allowing components to be substituted or deployed locally without affecting the overall system architecture or research methodology.

Node-RED^2^ was selected as a lightweight platform to support continuous monitoring of ECA interactions as shown in Supplementary Figure S3. The presented Node-RED flow was used to develop dashboard that received log metrics from the ECA via the Node-RED “HTTP in” node. These metrics are easily extendable, and the monitoring layer itself is replaceable without affecting the overall system architecture.

### 3.2 Methods

#### 3.2.1 Application of co-AI DBR methodology

The co-AI DBR methodology described in Section 2 was applied to design and evaluate an ECA platform and conversational pipeline in post-stroke rehabilitation context. This process included mock-up simulations, prompt engineering for the generation of 48 SDTs, and conversational pipeline emulation using these SDTs, followed by a *post hoc* quantitative evaluation of LLM response quality. A subsequent pilot validation of ECA’s physical instantiation in the Furhat robot with domain experts and patients’ relatives further refined aspects that SDT issue-centered patterns cannot fully represent and that SDT-driven emulations cannot assess. Using a questionnaire, we evaluated quantitative the speech-based dialogue interface, robustness to natural speech variability and responses to real user questions. Qualitative evaluations guided further refinement of the seed prompts, increasing the clinical grounding of the pipeline and reducing response overgeneralization.

The co-AI DBR methodology, including its five phases, SDT schema, session structure, and emulation workflow can be independently replicated. Prompt specifications are provided in Supplementary Appendices 1, 2. Formulas for evaluating linguistic properties, as well as the SUS and IMI questionnaires, are also provided. Pseudocode for detecting factual contradictions and bias is included in the Supplementary Material, along with an example SDT profile. Certain aspects of reproducibility in this study may vary across replications due to non-deterministic factors, such as the specific LLM versions, providers, inference parameters, and expert-in-the-loop qualitative and quantitative evaluations. As a result, the generated SDT profiles and numerical results assessing the quality of LLM responses may differ.

#### 3.2.2 Evaluation metrics and analysis framework

To evaluate the quality of the ECA platform and pipeline, we employed a multidimensional evaluation framework considering technical implementation, seed prompt relevance, LLM linguistic properties and factual consistency, and the ECA’s physical instantiation.

To evaluate the ECA platform performance, simulation logs generated using mock-ups provide two types of quantitative data: (1) time-based performance metrics, including processing and response times during simulations with mocked LLMs and Python child processes; and (2) completion metrics, including overhead from handling LLM errors, logged Python script errors, successful verification passes confirming that the mock-driven dialogue framework operated correctly across all test cases, and successful robot interaction passes. To evaluate the quality of the generated SDTs, we adopted the same two-step procedure described in Arannil et al. (2024). First, SDTs with identical sets of questions or concerns are examined to identify contradictions in their *Patterns*.*expected* or to detect duplicates, which are subsequently removed. Second, the remaining SDTs are checked for hallucinations (Mündler et al., 2023) by comparing expected keyword and phrase lists across shared issues. If two SDTs contain the same question, however have conflicting expected keywords or phrases, at least one response is non-factual, and the conflicting SDT(s) are removed.

To evaluate the quality of LLMs responses, we adopted a multidimensional evaluation framework that considers linguistic properties, factual consistency and demographic robustness. Linguistic quality was assessed via established lexical diversity metrics that are robust to text length, complemented by measures evaluating repetition. Factual consistency was evaluated by identifying contradictions between generated responses and structured patient profiles, while potential demographic bias was assessed through counterfactual sensitivity analysis. Attention was given to linguistic measures and lexical diversity. The metrics that are generally used are: Measure of Textual Lexical Diversity (MTLD), Moving-Average Type–Token Ratio (MATTR) and HD-D. Research provides evidence that they are very stable across different text lengths (Zenker and Kyle, 2021). We also used MAAS and Yule’s K indices (McCarthy and Jarvis, 2010) and hapax legomena rate (HLR) (Pierrehumbert and Granell, 2018) as other techniques for assessing lexical diversity.

MTLD (Equation 1) measures the length of word sequences that maintain a minimum Type-Token Ratio (TTR). It is one of the best metrics for LLM lexical diversity.

$$\text{MTLD}=\frac{\text{Total}\;\text{number}\;\text{of}\;\text{tokens}}{\text{Number}\;\text{of}\;\text{factors}}$$ (1)

where “factor” is reached when: $\text{TTR}=\frac{V}{N}\leq 0.71$ , V is the number of types (the unique words in the text) and N is the number of tokens (or the total number of words in a text, including repetition).

The MAATR (Equation 2) captures local lexical variation and is used in many recent NLP and computational linguistics studies.

$$\text{MaTTR}=\frac{1}{K}\sum_{i=1}^{K}\frac{V_{i}}{W}$$ (2)

where W is the window size (in tokens), V_i_ is the number of types in window *I* and K is the number of windows.

The HD-D (Equation 3) is resistant to noise and works even with moderate text lengths. It is often recommended for corpora of varied size.

$$\text{HD}‐D=\frac{1}{n}\sum_{i=1}^{V}\left[1-\frac{\frac{\left(N-f_{i}\right)}{n}}{\frac{\left(N\right)}{n}}\right]$$ (3)

where N is the total number of tokens, V is the total types, f_i_ is frequency of type *i*, *n* is the sample size (commonly n = 42).

MAAS (Equation 4) captures unique lexical information and often shows strong agreement with MTLD.

$$\text{MAAS}=\frac{\log N-\log V}{{\left(\log N\right)}^{2}}$$ (4)

where N is the total number of tokens, V is number of types and the logarithm is usually with base 10 or natural.

Yule’s K (Equation 5) measures repetition. When K is high the text is repetitive, whereas low K means diverse vocabulary.

$$K=10^{4}\left(\frac{\sum_{i=1}^{V}f_{i}^{2}-N}{N^{2}}\right)$$ (5)

where f_i_ = frequency of type i and N = ∑f_i_.

The hapax legomena rate (Equation 6) measures the proportion of words that occur only once. Pierrehumbert and Granell (2018) show that a good indicator of the productivity of a morpheme is the number of words containing it that occur exactly once.

$$\text{HLR}=\frac{V}{N}$$ (6)

where V is the number of words that occur exactly once and N is the total number of word tokens in the text. The ratio of hapax legomena to the type count increases for longer texts according to Honore in Malvern et al. (2004).

In addition to linguistic measures, we evaluated factual contradictions between generated responses and structured patient profiles. Unlike prior work that treats factual consistency as entailment against unstructured references (Or et al., 2022), we assess grounded consistency against structured patient traits, framing factual errors as record-level rather than textual contradictions. We used a rule-based, attribute-level verification procedure for detection of factual contradictions between generated responses and structured patient profiles using attribute-level verification prioritizing interpretability and reproducibility in line with Kryscinski et al. (2020). Details of this procedure are provided in the Supplementary Figure S1. To assess potential demographic bias, we performed a counterfactual sensitivity analysis following the approach in Nadeem et al. (2021), swapping gender-coded terms in patient issue-centered patterns while keeping the model’s response fixed (see pseudocode in Supplementary Figure S2).

To evaluate Furhat as a physical embodiment of the ECA based on LLMs for post-stroke rehabilitation, pilot experiments were conducted with domain experts and family members involved in post-stroke recovery. Using а questionnaire, participants assessed the system’s usability, safety, feasibility, and rehabilitative potential, as well as the effectiveness of the voice-based interface and the perceived realism of the dialog interactions. The questionnaire consisted of two parts: (1) System Usability Scale (SUS) (Brooke, 1996) with 10 Likert-scale items evaluating perceived usability, and standard SUS scoring procedure where higher scores indicate higher perceived usability of ECA; and (2) an adapted short version for Intrinsic Motivation Inventory (IMI)^3^ with 10 Likert-scale items assessing engagement, motivation and interaction quality, where higher values indicate stronger intrinsic motivation and engagement during interaction. Questions can be seen in the (Supplementary Tables S2, S4).

### 3.3 Participants

Fifteen participants (*12 F; 3 M*), aged 26–67 years (*M* ∼ 46.8), took part in the pilot human validation, including nine domain experts and six family members of patients recovering from stroke. The family members first completed a structured form to construct a virtual patient profile following the schema in Supplementary Appendix 1. The demographic and clinical characteristics collected from the patient profiles were as follows: *ID1{age: 58, gender: M, stroke type: Spinal, stage: Early}, ID2{age: 76, gender: M, stroke type: Hemorrhagic, stage: Early}, ID3{age: 59, gender: M, stroke type: Ischemic, stage: Early}, ID4 {age: 61, gender: M, stroke type: Ischemic, stage: Early}, ID5 {age: 54, gender: M, stroke type: Ischemic, stage: Late}, ID6 {age: 50, gender: F, stroke type: Ischemic, stage: Early}*. Before the session with the Furhat robot, the patient profile was inserted into the ECA prompt for LLM fine-tuning. The ECA instructions can be seen in Supplementary Appendix 2.

For patients ID3 and ID4, who were in the early stages following an ischemic stroke, a preprepared example of a weekly home-based rehabilitation plan was added to the ECA prompt to provide a more concrete and clinically grounded context. The plan for ID3 contains exercises training the affected limbs and trunk, while for ID4 - interventions addressing speech deficit and dysarthria. We asked participants to prepare 3–5 example questions in advance reflecting the types of issues, needs for assistance and daily inquiries they experience. Then they started the conversation with the ECA, which had been instructed in its prompt to end each response with a contextually relevant question. Participants could change the topic at any time. Sessions were open-ended, however typically lasted 20–30 min.

**FIGURE 2:**
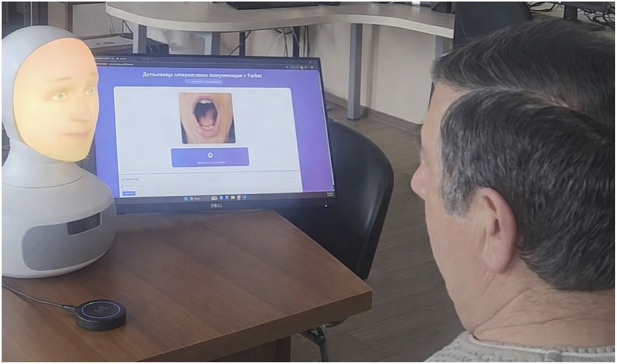
Experimental set-up.

The research experimental procedure and the online study was preregistered and approved by the Ethics Committee of the IR-BAS with protocol N13/20.01.2026. The experimental set-up is presented in Figure 2.

### 3.4 Results

**FIGURE 3:**
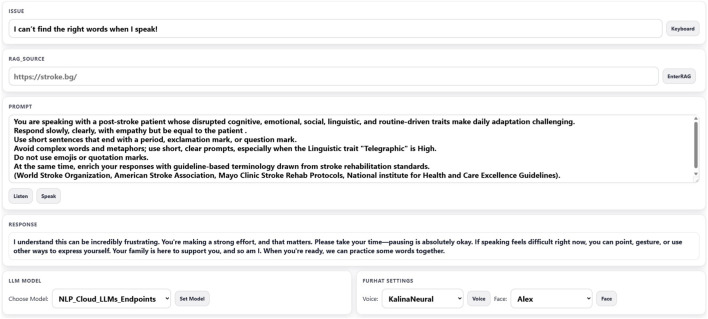
ECA platform.

The findings in Phase 1 resulted in different prototypes of the ECA platform, which led to the current design and implementation. The initial prototype was built using cloud APIs for natural language processing and generation, with a Node.js Express backend handling API endpoint and running Python scripts as child processes to control Furhat robot via Realtime APIs over a WebSocket connection. An internal and external SQLite database stored contextual data. Dashboard was developed in the Node-RED platform (Supplementary Figure S3). In subsequent iterations, the ECA web-based frontend (Figure 3) allowed configuration of question modalities, virtual or robot settings (voice, facial expressions) and the model of LLM. The platform was enhanced to support Retrieval-Augmented Generation (RAG), which connects the LLM to external databases or sources to automate information retrieval with ground-truth data. The main input to ECA is voice-based, however for nonverbal patients, a keyboard or picture board could be opened on external display to support Augmentative and Alternative Communication (AAC). This display was also used to support rehabilitation by presenting files, images or short videos synchronized with Furhat’s speech. This ECA can provide patient-specific visual guidance for complex motor exercises, object recognition for sensory deficits, and oral-motor sequences for apraxia and dysarthria, according to the media links in the augmented weekly plan. When such links exists, LLM is instructed to include them in the response with a keyword “source” at contextually relevant positions, followed by a structured object, e.g., *{“url”: “* https://youtu.be/ZKR1nOtCNKU?t=452 *”, “time”: “7:33–8:08”}*. Then, proposing a novel event-based trigger mechanism initiated from the LLM-generated response, media content is dynamically displayed on the screen during Furhat’s speech. Before the platform backend forwards the response to the Furhat Realtime API Python library, a middleware function parses the text and schedules each action based on the associated object. As the robot begins text-to-speech, the system continuously monitors upcoming tokens. When the keyword “source” is detected, instead of having the robot pronounce it, the system extracts the subsequent object and converts it into an event. If the object refers to a video, the specified clip is played on the display for the defined time interval. If the object refers to an image, the image is rendered and remains visible until the relevant segment of the utterance is completed.

The findings in Phase 2, guided subsequent unit and integration testing of the ECA platform with Jest, a JavaScript testing framework for Node.js. Mock development employed the *jest.mock*() function to automatically mock the *axios* module, returning test data via *mockResolvedValue* and simulating dialogues through a *mockDialog* object. Express app instance needs to be exported via *module.exports = app*, which enables backend components and test frameworks to import it directly, while the *require.main = = = module* condition ensures the server initiates only when executed as the primary entry point. Thus, we tested backend components, such as request handling, text generation with NLPCloud or BgGPT API, SQLite message storage, and dialogue system child processes, using Jest mocks to simulate HTTP requests and LLM responses without making external API calls, and verify python script execution without requiring the physical hardware of the robot. Python scripts that send commands to the Furhat robot for text-to-speech, listening or facial expressions were also mocked to simulate remote robot control via WebSocket. Server testing results of routes, middleware and external APIs: Jest checked 30 files, found 4 test files (including those using NLP Cloud) matching the specified patterns. The Furhat interaction passed successfully, including full interaction simulation with mocked LLM and Python (0.078 s), handling LLM failures (0.006 s), and logging Python script errors (0.055 s), with the full suite completing in 1.374 s. Then we generated a mock script into tests to simulate both linear dialogs and multi-turn, context-aware conversations without calling any API. All tests passed successfully in 0.284 s, confirming that the mock-driven dialog framework behaves as expected for both linear (4 ms) and context-aware (2 ms) conversations.

The result findings in Phase 3, are the development of seed data generation prompt how to instruct genAI to create SDTs for post-stroke patients, and a script generator that produces 10 user-utterance sessions per SDT over a 1-month period, designed to reflect gradual rehabilitation improvement. The prompt was generated across several iterations and is presented in Supplementary Data S1. To assess the SDT relevance, we performed two-step evaluation process. First, SDTs with identical sets of questions/concerns that have contradiction in their *Patterns*.*expected* or identified as duplicates to drop. Next, the remaining SDTs had been checked for hallucinations (Mündler et al., 2023) by comparing expected keyword lists for shared issues, i.e., if two different SDTs share the same questions however their expected keyword/phrase lists conflict, at least one of the conflicting responses must be non-factual and need to be dropped. The initial set of SDTs was assessed, and 15 SDTs were removed due to duplication or contradictions in questions relative to the patient profile. For instance, SDT42 was defined with ‘limited mobility’, however concern like “I cannot find the right words” had been generated. Thus, in the next iteration we asked within the seed prompt for clinically realistic questions or concerns, reflecting personal and clinical profiles, with expected responses according ten keywords aligned with the SDT’s profiles and dynamic states. We evaluated the logic of issues with respect to expected keywords and their categories. In the next refinement, we added the recovery goals, such as improving speech, regaining confidence, and returning to work, and enriched the patterns with clinical terminology and lexical richness grounded in stroke rehabilitation guidelines from World Stroke Organization resources^4^, National institute for Health and Care Excellence Guidelines^5^, American Stroke Association^6^, Mayo Clinic Stroke Rehab Protocols^7^, The Taking Charge After Stroke protocol (Fu et al., 2020), etc.

To enable tracking of patient status, dynamic state fields, such as mood, communication, mobility, fatigue, and progress history, were formalized. To ensure that the ECA could mimic the gradual rehabilitation progress represented in the SDTs, we developed a prompt_generator script that creates user utterances relevant to each *Patterns*.*expected* meaning. We improved realism by linking each prompt’s “category” (e.g., “Mobility”) to a corresponding set of questions. By mapping between SDT categories and seeds utterances, the script generates 10 sessions per SDT over a 1-month period, reflecting gradual improvement, i.e., early sessions emphasize fatigue or speech difficulties, while later sessions show more successful outcomes.

The findings in Phase 4, are the iterative emulations of the ECA pipeline with SDTs, conducted using Puppeteer - a Node.js library that enables programmatic control of Chrome-based browsers for automated testing, as well as evaluating the ECA pipeline for the realism of LLM-generated responses, along with the dynamic state updates for each SDT over a one-month period, designed to reflect gradual rehabilitation progress. The prompt for ECA, how to act as a rehabilitation coach, was also iteratively refined and consisted of instructions for LLMs. The prompt used during the puppeteering for emulation of ECA with SDTs is shown in Supplementary Figure S4. During the iterative design, we included the personal and clinical profile, rehab goals, and enriched the prompt with clinical terminology and lexical richness grounded in stroke rehabilitation guidelines like in Phase 3. During ECA emulation, each SDT sends questions to the frontend and receives responses, with SDTs emulating realistic user interaction behaviors and the ECA functionalities connected to actual AI cloud services. Responses were programmatically assessed in real time based on metrics such as response time, task completion, and errors, for checking whether the LLM responses included the expected keywords from the SDT schema. SDT behavior is visualized using a Node-RED dashboard, enabling real-time monitoring of interaction patterns.

During puppeteering, a platform script updates the SDT’s dynamic state using user utterance injections generated in Phase 3 from 10 user-utterance sessions per SDT over a one-month period, logging both the state and session metrics. Currently, a simple heuristic models depression risk in stroke patients by linking fatigue to speech fluency: high fatigue slows cognitive and speech progress, whereas speech practice can improve mood however, slightly increases fatigue. Mood also affects communication, e.g., when mood falls below 0.3, speech improvements are reduced to reflect low motivation. Positive feedback loops are included, as improvements in speech slightly enhance mood, reinforcing patient confidence and overall rehabilitation progress. Based on LLM analysis, the model was refined so that each session follows a continuous context rather than jumping between unrelated issues. A session_context variable ensures all lines remain within the same symptom or concern domain. Sessions begin with a clear main issue, and subsequent sentences use transitions referencing the previous line, creating logical flow. Statements progress from *issue - > impact - > emotion - > reflection - > small improvement*, with transitional phrases maintaining continuity. Unrelated symptom domains are no longer mixed, and positive reflections are placed at the end.

A demonstration showcasing the operational validation of the virtual ECA is available at^8^. After completing 10 sessions across all 48 SDTs, the relevance of the LLM-generated responses was ssesed. Interaction logs, containing user personal and clinical profiles, questions/concerns, and LLM responses, were analyzed using selected linguistic metrics. As we can see from the descriptive statistics (Table 1), the mean value of MAAS (0.016) is very low, meaning the vocabulary is consistently diverse across the responses. Since lower MAAS values correspond to higher lexical diversity, these results suggest that the model uses naturally varied vocabulary with no signs of fixed templates or constrained lexical patterns. MATTR measures local vocabulary richness, and the mean value is 0.818 which reflects rich vocabulary. It is diverse throughout the responses. This demonstrates that the model avoids repetition and uses flexible phrasing. MTLD is one of the most robust metrics for evaluating global lexical diversity. The observed mean value of around 93.86 indicates high lexical variety. These results show no evidence of formulaic structure across the text. The mean score for HD-D is 0.83, indicating the model’s vocabulary richness is consistent even across responses of different lengths. Repetition is another important dimension of lexical diversity. Yule’s K measures repetition, so when the values are lower, that leads to better diversity. The scores range from 0 to around 244, which suggests low to high repetition which is explainable since LLMs tend to be repetitive especially through consecutive iterations and in longer generated texts (Guo et al., 2024). The mean value for that parameter is around 115, which shows that moderate repetition occurs in responses. Taken together, these findings show that the model is well-balanced in its lexical behavior. It uses wide and varied vocabulary, maintains strong diversity across responses, but sometimes uses repetitive or templated patterns.

**TABLE 1:** Descriptive statistics of lexical diversity metrics.

Statistical descriptors	MAAS	MATTR_50	MTLD	HD-D	YULES_K
Count	2905	2905	2869	2905	2905
Mean	0.016	0.818	93.859	0.833	115.421
std	0.003	0.038	19.498	0.031	27.717
min	0.000	0.682	43.687	0.717	0.000
25%	0.015	0.796	80.146	0.815	99.584
50%	0.016	0.817	92.598	0.832	115.741
75%	0.018	0.837	105.876	0.848	131.281
max	0.027	1.000	166.017	1.000	244.137

**FIGURE 4:**
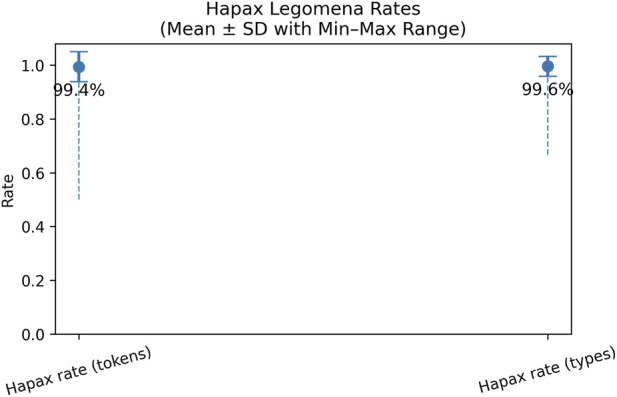
Hapax legomena rate.

For hapax legomena rate, we examined both the hapax rate per tokens and the hapax rate per types. On average, hapax legomena rates were consistently high across responses, with mean values of 99.4% for token-based hapaxes and 99.6% for type-based hapaxes. This reflects the short length and lexically varied nature of most generated responses, where most words occur only once. In comparison, Siddharthan in Siddharthan (2002) reported that in large natural language corpora almost half of all word types (49.8%) occur only once. However, the broad min–max ranges indicate substantial variability across individual outputs. Given the known sensitivity of hapax legomena to text length, these results align with more length-robust lexical diversity measures (Greenspahn, 1980). The results are shown in Figure 4.

**FIGURE 5:**
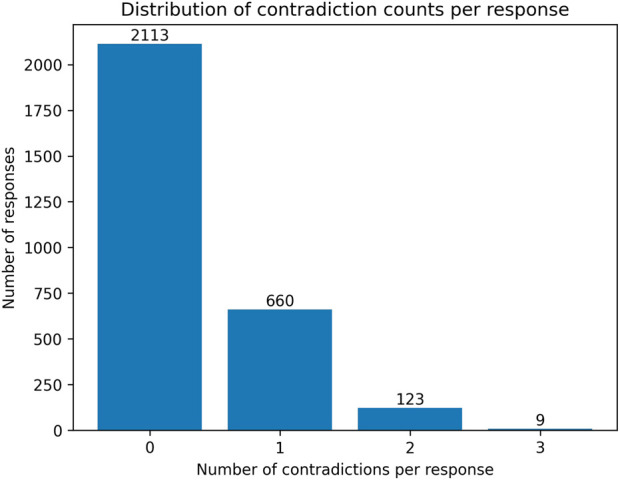
Distribution of model contradiction counts per response.

Figure 5 shows the distribution of contradiction counts per generated response and indicates that factual inconsistency primarily arises from single-attribute mismatches rather than widespread hallucination across multiple patient attributes. Most responses 2113 (out of 2905 generated responses) contain no detected contradictions, while 660 responses exhibit a single factual inconsistency. Responses with multiple contradictions are unusual, with 123 responses with 2 contradictions and 9 responses with 3 contradictions. This is fewer than 5% inconsistencies and suggests that when factual errors occur, they are typically isolated rather than pervasive.

**FIGURE 6:**
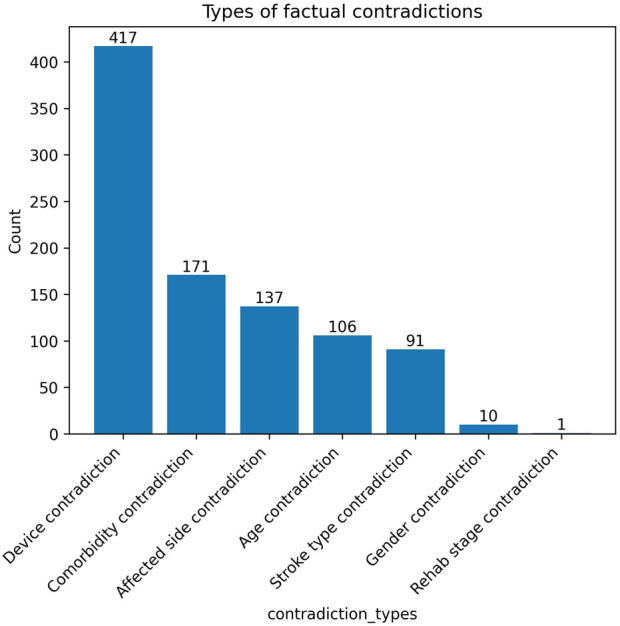
Distribution of factual contradiction types detected across generated responses.

Figure 6 shows the distribution of factual contradiction types detected across LLM generated responses. The most frequent contradictions involve assistive device use, followed by comorbidities and affected hemisphere. Those contradiction types correspond to low-salience, probabilistic or prototypical clinical attributes, which LLMs tend to fill in by default when generating patient-like narratives. In terms of assistive device contradictions, this could reflect overgeneralization rather than random hallucination, since assistive devices are often highly prototypical. Regarding comorbidity contradictions, models trained on population-level distributions tend to insert typical conditions, reflecting population priors and occasionally overriding patient-specific constraints. Contradictions related to the affected side may occur because “left/right” is sensitive information, making the models more prone to confusion in longer responses. Age contradictions are relatively uncommon, as age is often approximated (e.g., “elderly” “in his 70s”) or mentioned indirectly. Stroke type contradictions are low relative to the total number of responses (91 instances, ∼3%), probably reflecting a default to prototypical stroke categories when the subtype is not central to the communicative goal of the response. In contrast, contradictions involving core demographic attributes such as gender and rehabilitation stage are rare. This pattern indicates that factual errors predominantly arise in secondary or auxiliary clinical details rather than in primary patient characteristics. Finally, the prevalence of device-related contradictions suggests a tendency of the model to assume prototypical stroke recovery scenarios, even when such details are absent from the patient profile.

Following the analysis of the number and types of contradictions, we examined their distribution across the language models used to generate SDTs (Table 2). Grok-3 exhibited the highest contradiction rate (40%, corresponding to 8 contradictory responses). However, this result is based on a small sample of only 20 responses, as the remaining generated SDTs were excluded during Phase 3. Among the models that generated a larger number of SDTs, and therefore a larger pool of responses, Gemini-2.5-Pro exhibited the highest contradiction rate (33.1% - 148 contradictory responses), followed by DeepSeek-V3.2 (29.6% - 353 contradictory responses). ChatGPT-5.0 showed the lowest contradiction rate within this group (22.7% - 283 contradictory responses). Overall, the analysis suggests that factual consistency varies across the LLMs used to generate SDTs. These findings indicate that differences in model architecture or generation strategies can influence a model’s ability to maintain factual consistency in patient-specific scenarios.

**TABLE 2:** Contradiction statistics by language model.

LLM	Number of responses	Contradictory responses	Contradiction rate (%)	Mean contradictions per response
ChatGPT-5.0	1244	283	22.749	0.265
DeepSeek-V3.2	1194	353	29.564	0.344
Gemini-2.5-Pro	447	148	33.110	0.403
Grok-3	20	8	40.0	0.60

**FIGURE 7:**
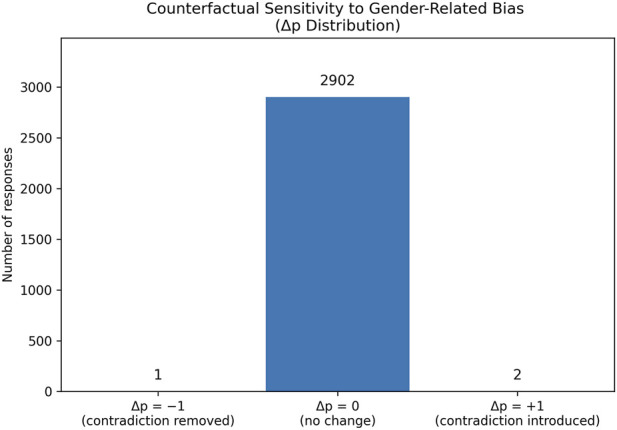
Counterfactual sensitivity analysis for gender-related bias.

In the counterfactual sensitivity analysis for gender-related bias (Figure 7), from 2905 generated responses only 47 issue prompts contained explicit gender-coded terms, indicating that gender framing was rare in the evaluated dataset. The mean contradiction rate for original issues (*p* contra = 0.2726) and for gender-swapped counterfactual issues almost the same value (*p* contra = 0.2729). The absolute mean change in contradiction probability was insignificantly small (∣Δp∣mean = 0.0010), indicating no systematic effect of gender framing on factual consistency. Non-zero Δ*p* values occurred only in a small number of cases, reflecting isolated edge cases rather than a consistent pattern. These results indicate minimal sensitivity to demographic framing under the evaluated conditions. However, because explicit gender cues were infrequent in the dataset, these findings should be interpreted as evidence of robustness to gender framing rather than as a comprehensive assessment of gender bias.

The findings in Phase 5, are based on reviews, tests, and questionnaires conducted with both the virtual and physical embodiments of the ECA platform and its conversational pipeline by a multidisciplinary expert group. It included three members affiliated with the Association for Stroke and Aphasia (a non-profit organization supporting post-stroke patients and their families), as well as experts from social and integration centers: three social science specialists, two psychologists, and four speech and language therapists. The experts first assessed the contextual realism of the synthetically generated profiles and issue patterns for interaction with the ECA, then they reviewed the virtual ECA’s operation and analyzed the logged LLM-generated responses. Overall, the technical implementation was highly valued. They confirmed that the AI-generated virtual patient representations successfully emulated the full operational ECA pipeline, including both frontend interactions and backend endpoints, without noticeable response delays. Experts highlighted the potential of the ECA framework as a training tool for junior therapists, particularly in delivering psychological and speech-language support for post-stroke patients and underlined its usefulness for telepractice. After final iteration, experts confirmed that the responses generated aligned with clinically validated guidelines and found them logical, relevant, and appropriate to session objectives, providing emotional support and psychological guidance. Expert critique guided successive system refinements during iterations in Phases 3 and 4. Initially, they observed that, although the model’s language was rich and expressive, contextual realism was limited and not consistently aligned with the user’s developmental or cognitive profile. They also noted repetitive phrasing and recommended stronger integration of the user’s individualized clinical context, cognitive state, and emotional state to enhance the psychological relevance and supportive quality of the dialogue. Experts also highlighted that terms such as “compassionate” and similar expressions show high concern and compassionate, however the patient should feel equal, and the ECA should express confidence and reproduce a pleasant sense of reassurance. Additionally, they emphasized that even when the context is well-established, the ECA should continuously monitor for contextual changes and signs of fatigue. Experts also stressed that when patients report positive changes (e.g., “I feel better today”), the ECA should not simply acknowledge the statement but systematically follow up with clarifying questions to explore underlying causes and reinforce therapeutic insight, such as assessing whether the interaction itself contributes to increased confidence and wellbeing.

In the pilot human deployment, experts further validated the Furhat robot as the physical instantiation of the ECA and, based on real user interactions via the speech-based interface, assessed the conversational pipeline performance, usability, and the acceptance of LLM-generated responses. Experts tested the ECA using one of the personal profiles collected from a patient’s family members or observed the ECA operating in its functional mode. They reassessed whether the ECA’s language remained consistent with established rehabilitation guidelines and evaluated its alignment with patients’ personal and clinical profiles, emotional tone, system performance, usability, and user acceptance of the LLM-generated responses to real questions. Experts noted that the ECA often presents multiple clinical terms within a single response, which may be difficult for patients or family members to understand. Such terms should be explained in accessible language. They also highlighted that personalization is insufficient and recommended complementing the patient profile by embedding the rehabilitation plan, including clear, step-by-step descriptions of each physical or speech-language exercise. Speech and language therapists recommended using the Furhat display to complement rehabilitation by presenting illustrative images and videos during therapy, as most speech-language interventions require real-time visualizations. For example, object pictures can support patients with sensory deficits or expressive aphasia, while video demonstrations of tongue massages or oral motor sequences can aid in the treatment of apraxia and dysarthria. Based on these discussions, we refined the SDT seed-guided prompt to incorporate the patient’s rehabilitation plan, including detailed descriptions of all exercises for both functional and speech-language therapy. The refined prompt used during the pilot human validation of the physical ECA is provided in Supplementary Appendix 2. An example for weekly home-based rehabilitation plan, which can be incorporated into the prompt as a PDF document via RAG, is provided in Supplementary Data S2. The plan includes detailed descriptions of all rehabilitation exercises, supplemented with media content, and was successfully tested to reduce generic responses. During the final two sessions with relatives, experts evaluated the responses to their questions as more concrete and useful. A notable improvement identified by experts was the integration of the external monitor with media content during the Furhat speech. As a key contribution, professionals found that the ECA enables rehabilitation to be structured into shorter sessions distributed throughout the day, in contrast to traditional rehabilitation clinics, which typically rely on a single continuous 1-h session.

Experts suggested further enhancements to the ECA platform and conversational pipeline, emphasizing that: (1) the ECA should communicate with confidence and convey a reassuring and pleasant tone, particularly when presenting complex or potentially distressing information, in order to maintain user comfort and reduce emotional pressure; (2) Initial session before each session, patient’s own words and phrases, prompt them to tell a story to assess their vocabulary, and detect the patient’s current emotional state, clarifying questions, discovering fragmented speech. It should include clarifying questions that guide toward the specific problem; (3) Discovering different symptoms of fatigue, such as *“losing the line of thoughts”*, because experience-dependent neuroplasticity requires concentration and energy, and the brain tires and loses focus. This appears as fragmented speech, or words like *“I’ll think about it”*.

After completing six sessions with relatives, we reevaluated the realism of the responses generated by the LLMs. Overall, the generated responses to real participants’ questions exhibit high lexical diversity across all evaluated metrics (Table 3). MTLD values are consistently high (Mean ∼134), indicating that the text maintains a high type–token ratio and robust lexical variation that is largely independent of response length. In comparison, the responses to virtual participants’ questions show a lower mean MTLD value of around 93.86, suggesting that the lexical diversity increased in the new response set. This pattern indicates that the generated texts maintain lexical diversity over longer passages, rather than displaying primarily local variation. Both MATTR and HD-D likewise show high average values (Means ∼0.88 and ∼0.90, respectively), reflecting strong local lexical variability. This confirms that these patterns are stable even for relatively short responses. The corresponding values for the responses to virtual participants’ questions are similar–mean MATTR of around 0.82 and mean HD-D of around, which suggests comparable local lexical variation and richness. Repetition-based measures provide a complementary perspective on these findings. The low mean MAAS value (Mean ∼0.009) further supports the presence of rich vocabulary usage, while Yule’s K mean ∼66.80 indicates moderate repetition driven by a limited set of frequently reused terms. The MAAS value of the virtual participants is almost identical (Mean ∼0.015), suggesting comparable levels of global lexical diversity. However, the Yule’s mean value in the responses to virtual participants’ questions is twice as big, around 115.42.

**TABLE 3:** Descriptive statistics of lexical diversity metrics.

Statistical descriptors	MAAS	MATTR_50	MTLD	HD-D	YULES_K
Count	121	121	119	121	121
Mean	0.009	0.881	134.091	0.895	66.801
Std	0.004	0.053	65.014	0.042	37.695
Min	0.000	0.749	55.155	0.781	0.000
25%	0.007	0.847	96.254	0.872	42.008
50%	0.008	0.889	121.816	0.902	57.455
75%	0.011	0.920	156.973	0.924	85.440
Max	0.019	1.000	591.500	1.000	179.063

**FIGURE 8:**
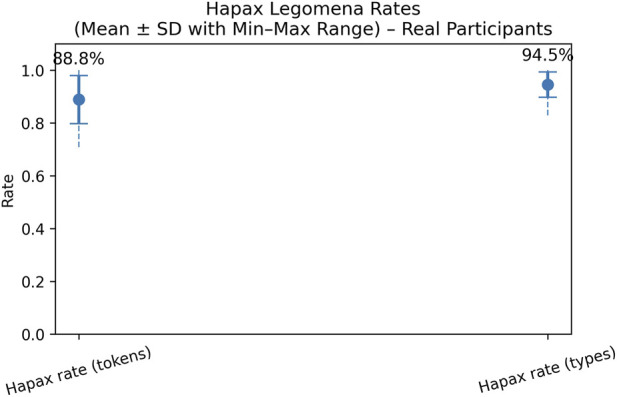
Hapax legomena rate.

Figure 8 shows high hapax legomena rates for the LLM-responses to real-participants, with a mean token-based hapax rate of 88.8% and a mean type-based hapax rate of 94.5%. This indicates that most lexical items in individual responses occur only once and that responses given to the real participants are lexically diverse. Compared to the responses to virtual participants’ questions, those to real participants’ questions show lower hapax legomena rates, particularly for the token-based measure (88.8% versus around 99.4% in synthetic data). The difference is smaller for type-based hapax rates, which decrease from 99.6% with virtual patients to 94.5% with real participants. Overall, these findings indicate a bit greater repetition in the responses generated with the real participants, which may be due to the specific weekly rehabilitation plans.

**FIGURE 9:**
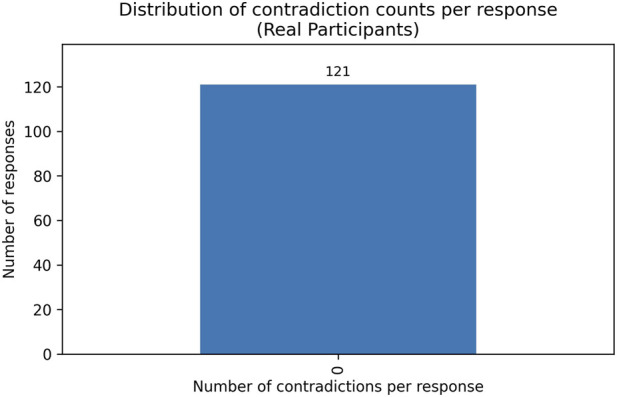
Distribution of factual contradiction types detected across generated responses.

Figure 9 shows the distribution of contradiction counts per generated responses to real participants’ questions. The distribution shows that none of the responses produced contained rule-detected factual contradictions with the corresponding patient profiles. All responses were fully consistent with the structured attributes, indicating that, unlike responses generated to virtual patients, those with refined ECA prompt and responses to real questions reliably adhere to patient-specific constraints.

**FIGURE 10:**
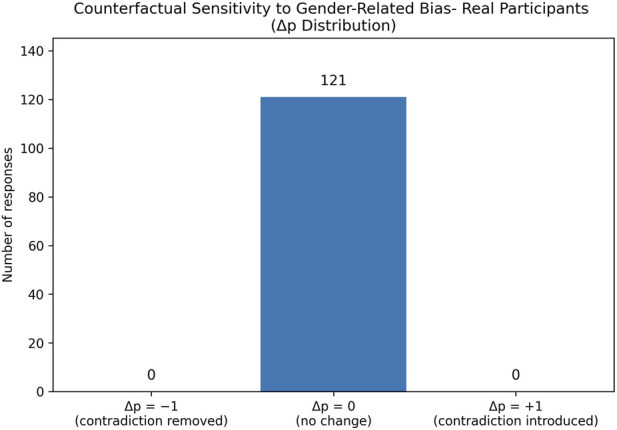
Counterfactual sensitivity analysis for gender-related bias.

Figure 10 shows the distribution of Δp values from the gender counterfactual sensitivity analysis for the real participant data. All responses fall into the Δp = 0 category, indicating that swapping gender-coded terms in the issue prompts neither introduced nor removed any factual contradictions. This result shows complete invariance of contradiction behavior to gender framing in the evaluated responses, providing strong evidence that the model’s factual consistency is unaffected by gender-coded wording under these conditions.

To examine perceived usability of the Furhat robot as ECA, participants completed the SUS questionnaire with a 5-point Likert scale and group-level descriptive statistics were computed for relatives and experts (Table 4). Descriptive statistics revealed higher perceived usability among relatives (Mean = 86.67) compared to experts (Mean = 72.50). relatives demonstrated more consistent evaluations (SD = 4.92), whereas experts’ ratings showed substantial variability (SD = 19.88). A possible explanation for the little higher SUS scores reported by relatives could be their closer alignment with the intended end-user profile. As a result, they may evaluate usability, focusing on whether the system feels helpful, understandable and supportive rather than on technical or design constraints.

**TABLE 4:** Descriptive statistics of SUS total scores (relatives vs. experts).

Group	N	Mean	SD	Median	Min	Max
Relatives	6	86.667	4.916	87.5	80	92.5
Experts	9	72.500	19.882	75	40	100

Participants’ intrinsic motivation was evaluated using the IMI questionnaire with a 7-point Likert scale (Table 5). The descriptive statistics of IMI scores, calculated for relatives and experts, revealed high levels of intrinsic motivation in both groups (Relatives: Mean = 6.32; Experts: Mean = 6.12). Given that IMI scores range from 1 to 7, mean values above 6 indicate strong intrinsic motivation across all participants. Standard deviations were comparable and relatively low in both groups (Relatives: SD = 0.96; Experts: SD = 0.86), indicating that relatives and experts experienced the interaction as engaging, interesting and motivating.

**TABLE 5:** Descriptive statistics of IMI scores (relatives vs. experts).

Group	N	Mean	SD	Median	Min	Max
Relatives	6	6.317	0.962	6.65	4.4	6.9
Experts	9	6.122	0.864	6.5	5	7

Descriptive statistics by item and by group (experts and relatives) for SUS and IMI scores are presented in the (Supplementary Tables S2–S5).

Together with experts, we conducted risk assessments following the rules and methodology in Iliuță et al. (2024), in particular those in Section 4.1 (“Considerations Regarding Risks Associated to Digital Twins”) and 4.2 (“Evaluating Risks Associated with the Integration of Digital Twins in Systems Medicine”), where authors as primary high-risks categorized: corrupted inputs, data leakage, unauthorized access, system control loss, performance failures and vulnerabilities from updates or maintenance. Thus, we analyzed the generated SDTs and LLM responses for potential harm and extended the concept of model inaccuracies to explicitly include “hallucinations” (3.2), recognizing errors as a common risk for LLMs. We also identified patient fatigue or negative mood leading to disengagement. which may contribute to potential risk and include it (3.6) as an unintended consequence of the ECA conversational pipeline. Supplementary Table S1 presents a co-AI DBR risk assessment framework.

## 4 Discussion

The findings of this study demonstrate the effectiveness of the proposed co-AI DBR framework, in which human–AI co-research supports iterative knowledge building. Although AI, with its extensive expertise for generating and analyzing information, can support designers and evaluators by enhancing the assessment of software solutions and rehabilitation practices, expert judgment remains essential, as evaluation requires critical thinking, domain expertise, and ethical responsibility. Through the involvement of domain experts and their critical evaluation, the conceptualization of design principles for clinically grounded ECA as rehabilitation coach, as well as the generation of prompt-based insights, has been made possible.

Regarding the technical findings, the ECA platform originally integrated LLM-driven dialogue with SDTs to enable emulation-based testing of patient-centered interactions, and this approach was successfully transferred to the Furhat robot, which functioned as an LLM-driven ECA in post-stroke rehabilitation settings. Question comprehension was evaluated via the voice interface with Furhat and as expected, performance was excellent, reflecting the high accuracy of Furhat’s native automatic speech recognition. The positive participant evaluations are reflected in the low variability in IMI scores across both experts and relatives, suggesting that intrinsic motivation elicited by the interaction was robust across differences in user roles and levels of expertise. Similarly, the lower variability among relatives on the SUS scale indicates a shared perception of usability, whereas the wider range of expert ratings may reflect different professional expectations. Although ConvAI was embodied in a robotic head rather than a full-body agent, this did not appear to negatively influence how the ECA was perceived, behaved, or communicated, particularly among non-technical users.

Regarding the lexical diversity analysis of LLM generated responses to SDT questions, results show that the LLM consistently uses rich and varied vocabulary across all responses. The high scores for MTLD, MAATR and HD-D, as well as the low values of MAAS, confirm these findings. Yule’s K reveals moderate levels of repetition (Mean = 117.04), with a few more repetitive responses, probably because of the nature of emotional support content. Hapax legomena rates further support the model’s lexical richness: 54.9% of all tokens and 78% of types are hapaxes, aligning with established patterns in natural language corpora (Siddharthan, 2002). These results confirm minimal repetition, naturalistic vocabulary use and clinical realism. Overall, factual contradictions were relatively infrequent, most often involving secondary clinical details (e.g., assistive devices, comorbidities, affected hemisphere). These findings suggest that models tend to rely on prototypical or population-level assumptions when generating patient-like narratives. At the model level, contradiction rates varied substantially, implying that differences in model architecture or generation strategies affect factual consistency. Fine-tuning or applying constraint-based generation could potentially enhance patient-specific accuracy.

Regarding the lexical diversity analysis of LLM generated responses to real participants’ questions, results revealed differences compared with responses to virtual participants’ questions. This difference may arise from variations in the ECA prompts (Supplementary Figure S4 vs. Supplementary Appendix 2) and the use of LLM-generated synthetic questions during emulation instead of real participant questions and concerns. For this reason, we consider it important to include the participant questions to Furhat robot in Supplementary Data S3.

Overall, the results and discussion support a positive answer to our research question: How can integrating human–AI co-research and emulative SDTs within a co-AI DBR framework advance iterative knowledge building for the development of ECAs that provide personalized, clinically aligned, and contextually realistic rehabilitation support, while enabling systematic evaluation of LLM-driven interactions and safe, ethically grounded deployment? Finally, we derived the main design principles: (1) Human–AI co-research methodologies accelerate and facilitate the design and testing of ECAs; (2) Prompt engineering and emulative SDTs can be used to represent diverse patient profiles and generate contextually grounded rehabilitation interactions; (3) Mock-ups and emulative SDTs enable realistic evaluation of ECA interactions of frontend, backend, and LLM-based functionalities of the Furhat robot, acting as a post-stroke rehabilitation coach; (4) The ECA should promote equality and reassurance by confidently engaging patients in a personalized pre-session dialogue that leverages their own language to assess emotional state, vocabulary, and fragmented speech, thereby enabling coherent and logically connected session content; (5) The ECA primarily relies on voice input, however for nonverbal patients, an external display has to support AAC; (6) An event-based trigger mechanism initiated by the LLM-generated response is required to dynamically display rehabilitation multimedia content on the screen during Furhat’s speech; (7) ECA interactions should align with rehabilitation guidelines and ensure ongoing risk management.

The following subsections present the risk assessment and control mechanisms used in human–AI co-research during early-stage ECA design. Particular attention is given to phases 3 and 4, where we encountered challenges related to prompt engineering and LLM accuracy, and to Phase 5, where patient fatigue or negative mood may reduce engagement, potentially causing early session termination and poorer rehabilitation outcomes.

### 4.1 Risk assessment and mitigation in human–AI co-research in early-stage ECA design

#### 4.1.1 Risk management actions in Phase 1 - Problem analysis and initial ECA prototyping

- Relevant risks (risk IDs in brackets correspond to Supplementary Table S1):
  1. Corrupted/erroneous input capture for early data gathering (1.1), which affects the accuracy of stakeholder analyses, interaction pattern mapping, and the initial definition of patient needs. *Actions:* Input validation and confirmation prompt for early data gathering.
  2. Insufficient/biased training data during AI-powered stakeholder analysis (3.1). *Actions:* Performed clinician review to avoid early conceptual biases.
  3. Ethical/transparency risks (3.3): lack of model explainability and auditability during early conceptualization. If the logic behind how an agent produced a specific, potentially problematic test case is unclear, it is impossible to ethically or reliably diagnose the software system. *Actions:* Applied Explainable AI (XAI) protocols and Traceability Logging for the generative model. Ensured that all synthetic data and test outcomes are linked to the specific model version and parameters used for their creation.

#### 4.1.2 Risk management actions in Phase 2 - Iterative mock-up–driven architectural design for early ECA testing

- Relevant risks:
  1. Human-induced corruption, as faulty scripts or test inputs (1.1). *Actions:* Validation of mock input structures before automated test runs. Implemented static analysis and linting on all test scripts.
  2. Sensitive information leaks during data streaming (1.2). *Actions:* Applied Transport Layer Security protocol (TLS 1.3) with mutually authenticated certificates for secured channels.
  3. Log loss or alteration: Initial testing phases often lack robust logging infrastructure, risking data loss crucial for debugging (2.1). *Actions:* Applied immutable logging policy WORM (write once read many) ensuring logs are timestamped, indexed, and redundantly replicated.
  4. Software faults from human error: misconfiguration of mock components (e.g., incorrect error codes, response timing) that hide real system bugs or create false positives (2.3). *Actions:* Applied mock configuration peer review and versioning.
  5. Loss of control: incorrect configuration of mocked pipelines or infrastructure can distort system behavior (4.2). *Actions:* Implemented automated drift detection to ensure the mock environment matches the changes in the quality of software over time.

#### 4.1.3 Risk management actions in Phase 3 - AI co-design during SDT generation

- Relevant risks:
  1. Corrupted/invalid SDT-generated inputs (1.1)*. Actions:* Experts-in-the-loop review process to manage risks from the prompt_generator script when the generated set of questions or concerns was
  2. Biased or insufficient SDT data: the ground truth data used to train the ECA is skewed, incomplete, or fails to represent critical edge cases, leading to unreliable or discriminatory SDT profiles (3.1). *Actions:* Identified specific missing corner cases and fill those gaps by iterative seed prompt refinement, following the “gold-standard” pass conditions that every updated prompt must meet to demonstrate no degradation. Applied cross-model SDT generation to ensure creative consistency across genAI models.

For interoperability testing, 48 SDTs were generated using different LLMs: OpenAI (14) and Copilot (4) both using ChatGPT-5.0, Gemini (10) using Gemini-2.5-Pro, DeepSeek (18) using V3.2 and Grok using Grok-3 (2). We encountered a prompt-engineering challenge during the generation of 48 VPs simultaneously. Most of the genAI models tended to reuse a single generalized template per “issue” varying only the personal profile or category. This resulted in repetitive and insufficiently diverse outputs. However, when generating one or two SDTs the schema was followed strictly, and the genAI produced detailed outputs using varied sentence structures and templated elements, while maintaining personalized and clinically relevant content. To address this limitation, we generated the 48 VPs in smaller batches (two at a time) and subsequently concatenated the SDTs into a single JSON file.

3. AI inaccuracies/hallucinations: The LLMs used to generate the adaptive and behavioral profiles for the SDTs produce non-sensical, clinically impossible, or contradictory conversational patterns (hallucinations), making the resulting SDT unusable for realistic testing (3.2). *Actions:* Implement a Human-in-the-Loop Clinical Review process where prompts and generated behavioral outputs are validated and corrected before final approval. Use Retrieval-Augmented Generation (RAG) techniques to ground the AI output in specific and reliable clinical guidelines.

#### 4.1.4 Risk management actions in Phase 4 - Iterative emulation with SDTs of web interaction flows and backend logic to validate the ECA in context

- Relevant risks:
  1. Log loss or overwriting: Critical test execution data (ECA responses, SDT evaluations, timing data) is lost or overwritten, hindering the ability to trace system failures and performance bottlenecks (2.1). *Actions:* Implemented logs tagged with the SDT ID, test run ID, and timestamps for precise traceability.
  2. AI inaccuracies/hallucinations: the ECA provides an inaccurate or hallucinatory response to the SDT’s question, and the automated prompt testing system fails to properly detect and flag this clinical or linguistic failure (3.2). *Actions:* Analysis of performed descriptive statistics of lexical diversity metrics: MAAS; MATTR; MTLD; HD_D; Yules_K, Hapax legomena rate, factual contradictions, user-model hallucinations. Experts’ clinician validation for realism.
  3. Vulnerable API/model endpoints: The internal ECA APIs (endpoints) that the SDTs interact with during the emulated test are insecurely exposed to unauthenticated or malicious queries (3.4). *Actions:* API security measures to segment and isolate unwanted traffic and API call rate limiting for access control, along with continuous monitoring of query patterns from the SDT simulator to detect anomalous behavior (to be implemented).
  4. Human factors, misinterpretation and misuse (3.5). *Actions:* Human-in-the-loop oversight, user training on how to interpret the AI outputs that assist ECA dialog, safety-layer prompts. Performing lexical measures.
  5. Patient fatigue or negative mood may reduce engagement, causing early session termination and poorer rehab outcomes (3.6). *Actions:* Caregiver-in-the-loop supervision, real-time sentiment/emotion detection (from speech and facial cues), and biosensor-based monitoring dashboard for continuous assessment and timely intervention.
  6. Performance degradation under SDT load: Running multiple concurrent SDTs simulates high user traffic, causing the ECA system or the testing infrastructure itself (e.g., browser farm) to slow down, time out, or fail, invalidating the test results (4.1). *Actions:* Implement Load Benchmarking before full deployment. Use dynamic scaling for the testing infrastructure and monitor resource usage (CPU, RAM, network I/O) continuously. Establish clear Service Level Objectives (SLOs) for test execution 3.6time and pause/terminate runs that violate these SLOs to prevent cascading failure.

#### 4.1.5 Risk management actions in Phase 5 - Reflection and formulation of design principles

Since Phase 5 is specifically designed to validate the realism, context awareness, clinical alignment, and ethical compliance of the ECA architecture and pipeline with expert-in-the-loop, the most important risks are: (1.1), (2.2), (3.1), (3.2), (3.3), (3.5), (3.6), (4.3), and two newly identified risk derived during the expert evaluation concerning the conversational safety and therapeutic appropriateness (3.5), and (3.6) - the patient fatigue or negative mood leading to disengagement or even deterioration. Most of the required risk-management actions still need to be implemented, as they depend on establishing a methodology for Clinical Validity Checklist and Ethical Review Checklist to assess the ECA and SDTs. This methodology should exploit safety protocols, clinical rehabilitation guidelines and define the “gold-standard” pass conditions that every refinement must meet to ensure no degradation in clinical and ethical metrics before deployment.

The risks currently mitigated are: (1.1) Erroneous/corrupted input capture: misinterpretation of fragmented or aphasic speech; missed signs of fatigue. *Actions:* The evaluation dashboard must present concurrent multi-modal data (text, audio, system flags) to allow experts to validate the model’s interpretation against the STD input; (2.2) Unauthorized access to evaluation logs from SDT data. *Actions:* Pseudo anonymizing all log data has been implemented to strictly limit platform access when applied to physical DTs; (3.5) Human factors, misinterpretation and misuse (3.5). *Actions:* Combination of quantitative metrics with expert-driven qualitative assessments, thereby adhering to established gold standards for testing and evaluating LLMs in (Aparna et al., 2024): “Scalability of human evaluation is also crucial to wider adoption”. All remaining risks’ mitigations are subject to future work.

### 4.2 Limitations of co-AI DBR

Despite promising outcomes, this study has several limitations that may affect the generalizability of the findings. The iterative evaluations were conducted within limited experts and participants, within a national context, which may restrict the transferability of the results to other regions. Broader validation is needed to assess the ECA platform’s long-term impact and adoption.

During Phase 4 and human validation, a key limitation was that only LLaMA 3-70B was evaluated, since it was the only available option at the time. While not among the most advanced models, it allowed us to evaluate the ECA’s robustness across different LLMs. At present, the ECA achieves improved performance in responses when using more advanced models available from NLP Cloud provider: gpt-oss-120B.

### 4.3 Future design considerations

Future work should extend validation with real patients or their human digital twins (DTs), ensuring that the system’s recommendations and interaction designs are clinically relevant, effective and aligned with patient needs. Insights from SDTs seed generation can serve as guidance for modeling DTs of post-stroke patients. Furthermore, we will integrate all design recommendations from the experts to optimize interactions with post-stroke patients, focusing on improving LLM response time and extending the capabilities of the Furhat screen-based modality synchronized with Furhat speech events. These enhancements will support the AAC, enable demonstrations of rehabilitation exercises, and provide structured choices for user input. The validated SDT issues will be particularly useful for enhancing ECA interactions, enabling to handle two types of user input: (1) Unconstrained input, allowing free-form interaction via text, speech or other AAC methods, which may feel more natural, however often less preferred by users; (2) Constrained input, where individuals select from predefined options, thus minimizing errors and accommodating stroke survivors who may face cognitive or physical challenges. This aligns with research in Richards et al. (2023), noted that constrained, multiple-choice dialogue systems, such as TaCIA, help minimize errors and accommodate stroke survivors who may face cognitive or physical challenges in generating responses. While unconstrained input may feel more natural, research indicates that users often prefer structured choices, which guide responses and deliver more accurate and empathetic replies (Amini et al., 2013).

## 5 Conclusion

The integration of human–AI co-research within the co-AI DBR framework effectively supports iterative knowledge building for the development of ECAs. By generating synthetically digital twins, the framework enables modeling of diverse patient profiles and contextually rich rehabilitation scenarios, overcoming limited access to real users in early design stages. This approach provides a safe and controlled environment to test, refine, and validate LLM-powered ECA responses. Combining iterative quantitative evaluation with expert feedback ensures comprehensive and reliable assessment. LLM-based ECAs show potential to deliver personalized, context-aware informational and supportive functions for home-based rehabilitation, reduce depressive symptoms, and enhance independence in daily living, demonstrating the potential of co-AI DBR to advance rehabilitation technologies.

## Data Availability

The raw data supporting the conclusions of this article will be made available by the authors, without undue reservation.

## Footnotes

1
https://www.furhatrobotics.com

2
https://nodered.org/

3
https://db.arabpsychology.com/scales/intrinsic-motivation-inventory-imi

4
https://www.world-stroke.org/publications-and-resources/resources

5
https://www.nice.org.uk/guidance/ng236/chapter/Recommendations

6
https://www.stroke.org/en/help-and-support/resource-library/lets-talk-about-stroke/lifestyle-changes

7
https://www.mayoclinic.org/diseases-conditions/stroke/in-depth/stroke-rehabilitation/art-20045172

8
https://youtu.be/xsKlFaRtWzA
