# Supplementary material for: Human–AI co-research on design and evaluation of Embodied Conversational Agent in rehabilitation contexts
Source: Front Robot AI. 2026 Mar 26;13:1758391. doi: 10.3389/frobt.2026.1758391 (PMC13062796; doi:10.3389/frobt.2026.1758391)
Supplement: Supplementary file 1 [file DataSheet1.pdf]

Supplementary Material

1 Supplementary Figures

Rule-Based Factual Contradiction Detection Procedure

1. **Load input data and initialize variables**
    - Load the CSV file containing generated responses, issue prompts, and structured patient profiles.
    - Specify column names for the issue text and generated response.
    - Initialize empty fields to store contradiction flags, counts, and explanations.
2. **Preprocess textual input**
    - For each data entry, concatenate the issue prompt and generated response.
    - Convert the combined text to lowercase.
    - Handle missing or malformed values by converting them to empty strings.
3. **Extract structured patient attributes**
    - Retrieve patient attributes from the structured profile, including:
        - Age
        - Gender
        - Stroke type
        - Affected hemisphere
        - Rehabilitation stage
        - Assistive devices
        - Comorbidities
    - Convert list-like fields (e.g., assistive devices, comorbidities) into normalized sets of lowercase strings.
4. **Initialize contradiction tracking**
    - Create an empty list to store detected contradiction reasons for the current response.
5. **Check gender consistency**
    - Detect gender-related cues (e.g., "he", "she") in the generated text.
    - Flag a contradiction if the detected cue conflicts with the patient's recorded gender.
6. **Check age consistency**
    - Extract numeric age mentions from the generated text using pattern matching.
    - Flag a contradiction if any mentioned age differs from the patient's age by more than a predefined tolerance.
7. **Check stroke type consistency**
    - Identify mentions of stroke types using a predefined vocabulary.
    - Flag a contradiction if the mentioned stroke type does not match the patient's recorded stroke type.
8. **Check affected hemisphere consistency**
    - Detect mentions of "left" or "right" in the generated text.
    - Flag a contradiction if the mentioned hemisphere conflicts with the patient's affected side.
9. **Check rehabilitation stage consistency**
    - Detect rehabilitation stage terms (e.g., acute, subacute, chronic).
    - Flag a contradiction if the mentioned stage does not match the patient's recorded stage.
10. **Check assistive device consistency**
    - Identify mentions of assistive devices in the generated text.
    - Flag a contradiction if devices are mentioned that are not present in the patient's profile.
11. **Check comorbidity consistency**
    - Identify mentions of common comorbidities using a predefined vocabulary.
    - Flag a contradiction if additional comorbidities are mentioned that are absent from the patient's profile.
12. **Aggregate contradiction results**
    - Set a contradiction flag if at least one contradiction is detected.

```
        o  Count the total number of contradictions.
        o  Concatenate all contradiction explanations into a single descriptive field.
  13. Save results
        o  Append contradiction indicators and explanations to the original dataset.
        o  Save the augmented dataset to an output CSV file.
```

Figure S1 | Rule-based procedure for detection of factual contradictions between generated responses and structured patient profiles using attribute-level verification

Algorithm: Counterfactual Sensitivity Analysis for Gender Framing

Input:

- Dataset D with columns: issue x, response y, patient profile P
- Gender swap rules S (e.g., he ↔ she, his ↔ her, male ↔ female)
- Rule-based contradiction detector $C(\cdot, P)$ returning $p_{\text{contra}} \in \{0,1\}$

Output:

- Augmented dataset D' with counterfactual issues, contradiction flags, and Δp
- Summary statistics of sensitivity to gender framing

Pseudocode

1. **Load data**
   - Read dataset D containing patient issues x, model responses y, and structured profiles P.
2. **Generate gender counterfactual issues**
   - For each record $r \in D$:
     - Detect whether x contains gender-coded terms.
     - Create a counterfactual issue x^{cf} by swapping gender-coded tokens using rules S.
     - Store x^{cf} and a boolean indicator hasGenderTerms(x).
3. **Evaluate contradictions for original issues**
   - For each record r:
     - Compute contradiction outcome for the original issue:
       $$p_{\text{contra}}(x, P) \leftarrow C(x \parallel y, P)$$
     - Store $p_{\text{contra}}(x, P)$ and the number of detected contradictions $n_{\text{contra}}(x, P)$.
4. **Evaluate contradictions for counterfactual issues**
   - For each record r:
     - Compute contradiction outcome for the counterfactual issue:
       $$p_{\text{contra}}(x^{cf}, P) \leftarrow C(x^{cf} \parallel y, P)$$
     - Store $p_{\text{contra}}(x^{cf}, P)$ and the number of detected contradictions $n_{\text{contra}}(x^{cf}, P)$.
5. **Compute counterfactual sensitivity metric**
   - For each original-counterfactual pair:
     $$\Delta p \leftarrow p_{\text{contra}}(x^{cf}, P) - p_{\text{contra}}(x, P)$$
   - Interpret:
     - $\Delta p = 0$: invariance to gender framing
     - $\Delta p \neq 0$: sensitivity to gender framing
6. **Aggregate summary statistics**
   - Compute:
     - Total number of samples N
     - Number of issues containing gender-coded terms
     - Mean contradiction rate for original issues:
       $$\bar{p}_{\text{contra}}(x, P)$$
     - Mean contradiction rate for counterfactual issues:
       $$\bar{p}_{\text{contra}}(x^{cf}, P)$$

Mean and absolute mean of Δp

     - Proportion of samples with $\Delta p \neq 0$
7. **Save outputs**

- Export augmented dataset D' including:
    - x^{cf}, hasGenderTerms(x),
    - $p_{\text{contra}}(x, P)$, $p_{\text{contra}}(x^{cf}, P)$,
    - $n_{\text{contra}}(x, P)$, $n_{\text{contra}}(x^{cf}, P)$,
    - Δp

Figure S2 | Pseudocode for counterfactual sensitivity analysis for gender framing: contradictions are evaluated on original and gender-swapped issues while keeping the patient profile and model response fixed; sensitivity is quantified via Δp

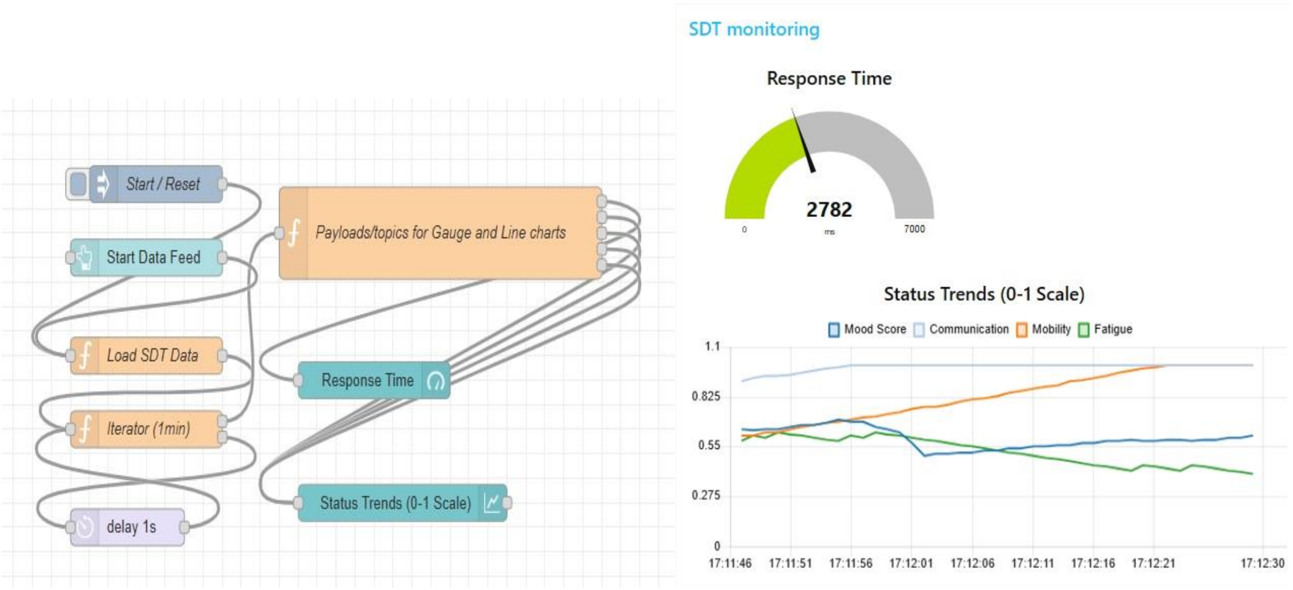

Figure S3 | Programming flow (left) and Dashboard (right) in Node-RED

```
You are speaking to a post-stroke patient who struggles with emotional regulation. Respond slowly, clearly and with empathy.
Use short sentences that end with a period, exclamation mark, or question mark. Avoid complex vocabulary and metaphors.
Do not use emojis or quotation marks.
Enrich your responses with guideline-based terminology drawn from stroke rehabilitation standards (World Stroke Organization, American Stroke Association, Mayo Clinic Stroke Rehab Protocols, National institute for Health and Care Excellence Guidelines).
When giving advice, naturally mention clinical terms such as:
- physiotherapy, occupational therapy, speech therapy
- energy conservation, neuroplasticity, constraint-induced therapy
- secondary stroke prevention, medication adherence
- adaptive equipment (shower chair, cane, walking aid), balance training
- emotional regulation strategies, cognitive rehabilitation, family education
Blend these terms in a conversational way so the patient understands the advice and feels supported.
Keep the tone compassionate and clear, however add realistic medical and rehabilitation vocabulary to align with clinical guidance while staying patient-centered.
Use both the personal and clinical profile below to tailor your responses:
```

Figure S4 |The prompt used during the puppeteering for emulation of ECA with SDTs

2 Supplementary Tables

Table S1 | co-AI DBR representation of risks, origins and management actions.

Layer	Risk	Origin	Description	Risk management actions
**1. Data Acquisition & Dissemination**	**1.1 Corrupted / erroneous input capture** PO - High, I – High.	Noisy audio, packet loss, user errors	Input channels resemble vulnerable DT sensors; faulty streams distort reasoning.	Input validation, consistency checks, confirmation prompts.
	**1.2 Sensitive information leaking during data streaming** PO - Medium, I – High.	Weak transport security	Unsecured channels risk may expose sensitive data as in DT data-flow risks.	TLS 1.3 with mutually authenti-cated client/server certificates
	**1.3 Compromised client device** PO - Medium, I - Medium.	Unsecured patient devices	Local-device compromise may inject altered data.	Client warnings, minimal edge processing, integrity checks.
**2. Data Management & Synchronization**	**2.1 Loss/alteration of logs** PO - Low, I - High.	Human error, misconfigurations	Matches DT data-loss risks: stored sessions may be overwritten/corrupted.	Backups, versioning, restore procedures.
	**2.2 Unauthorized DB access** PO - Medium, I – High.	Weak ID, lack of Multi-Factor Authen-tication (MFA)	Similar to DT unprotected DBs: adversaries may read/alter sensitive data.	Encryption, credential hardening, audit logs.
	**2.3 Software faults from human error** PO - Low, I - High.	Faulty scripts	Aligns with DT human-error data risks.	Script testing, peer review, protected tablespaces.
**3. Digital Model Layer & AI reasoning**	**3.1 Insufficient / biased training data** PO - Low/Medium, I - High.	Lack of clinical diversity	Comparable to DT “lack of training data”: reduces accuracy and generalizability.	Diverse datasets, clinician validation, periodic retraining, "gold-standard" pass conditions.
	**3.2 AI inaccuracy / hallucinations** PO - High, I – High.	Incomplete context in prompt; Incorrectly configured LLM parameters; Text genAI limitations.	DT model-accuracy analogue: faulty reasoning leads to misinterpretation. Errors in responses -wrong, missing, or contradictory information.	Measures: MAAS; MATTR; MTLD; HD_D;Yules_K, Hapax legomena rate, Factual contradictions. Experts’ validation for clinical realism.

	**3.3 Ethical / transparency risks** PO-Medium, I–Medium	Black-box behavior	Matches DT ethical transparency issues.	Explainability layer, consent, data minimization.
	**3.4 Vulnerable model endpoints** PO - Medium, I – High.	Unprotected APIs	Attackers may probe or manipulate model behavior.	Authentication, rate limits, HTTPS/TLS-protected endpoints, anomaly detection.
	**3.5 Human factors, misinterpretation and misuse** PO - High, I – High.	User misunderstanding, over-trust in AI outputs.	Clinicians or users may misinterpret or over-rely on LLM/DT outputs or disengage, affecting rehabilitation outcomes.	Human-in-the-loop oversight, user training, safety-layer prompts. Performing lexical measures.
	**3.6. Patient fatigue / negative mood leading to disengagement** PO - High, I – High.	Lack of supervision for patient fatigue or negative mood	Emotional distress or fatigue during ECA interactions may reduce a patient's motivation, lead to early session termination, prompt avoidance of rehabilitation, or eventually worsen therapeutic outcomes.	**Caregiver-in-the-loop monitoring**, sentiment detection from speech, emotion recognition by ECA, biosensors integrated into **dashboard** to alert clinician.
**4. Virtualization & Accessibility**	**4.1 Low system performance** PO - Medium, I – High.	High load, unoptimized inference	Mirrors DT latency/scalability issues affecting accessibility.	Autoscaling, load balancing, pipeline optimization.
	**4.2 Loss of control** PO - High, I – High.	Infrastructure technical or human/genAI errors in configuration or operation of the DT.	Mistakes in configuration, operation, or oversight result in mismanagement of the DT, thereby altering system behavior.	Encrypted channels, Mock-ups and emulative SDTs
	**4.3 Continuous update/maintenance issues** PO - Medium, I – High.	Cyber-attacks	Interruption of DT operations, introduction of vulnerabilities, and modification of security features.	Mock-ups and emulative SDTs for compatibility checks.

Table S2 | Descriptive statistics for SUS by item and by relatives.

**Item**	**N**	**Mean**	**SD**	**Median**	**Min**	**Max**
1. I would like to use the robotic personal assistant (Furhat) frequently.	6	4.66	0.51	5	4	5
2. I found the robotic personal assistant unnecessarily complex to use.	6	1	0	1	1	1
3. The robotic personal assistant was easy to use.	6	4.5	0.83	5	3	5

4. I would need assistance from someone else to be able to use the robotic personal assistant.	6	1.16	0.40	1	1	2
5. I believe that all parts of the session were successful.	6	4.5	0.54	4.5	4	5
6. The robotic personal assistant was confusing or behaved inconsistently.	6	2.5	1.37	2	1	5
7. I think that most people would learn to use the robotic personal assistant very quickly.	6	5	0	5	5	5
8. I found the robotic personal assistant very strange or difficult to interact with.	6	1.16	0.40	1	1	2
9. I felt confident while using the robotic personal assistant.	6	4.66	0.51	5	4	5
10. I had to learn a lot of new things before I could start using the robotic personal assistant.	6	2.83	1.83	2.5	1	5

Table S3 | Descriptive statistics for SUS scores, by item and by experts

**Item**	**N**	**Me an**	**SD**	**Medi an**	**Min**	**Max**
1. I would like to use the robotic personal assistant (Furhat) frequently.	9	4.22	0.97	5	3	5
2. I found the robotic personal assistant unnecessarily complex to use.	9	1.88	0.92	2	1	4
3. The robotic personal assistant was easy to use.	9	3.66	1.11	4	2	5
4. I would need assistance from someone else to be able to use the robotic personal assistant.	9	2.66	1.32	2	1	4
5. I believe that all parts of the session were successful.	9	3.77	1.20	4	1	5
6. The robotic personal assistant was confusing or behaved inconsistently.	9	1.8	0.6	2	1	3
7. I think that most people would learn to use the robotic personal assistant very quickly.	9	4.11	0.92	4	3	5
8. I found the robotic personal assistant very strange or difficult to interact with.	9	1.77		2	1	4
9. I felt confident while using the robotic personal assistant.	9	3.77	1.09	4	2	5
10. I had to learn a lot of new things before I could start using the robotic personal assistant.	9	2.33	1.49	2	1	5

Table S4 | Descriptive statistics for IMI scores, by item and by relatives.

Item	N	Mean	SD	Median	Min	Max
1. I enjoyed interacting with the robotic personal assistant.	6	6.33	0.81	6.5	5	7
2. The information and support provided by the robotic personal assistant were useful.	6	6.33	1.63	7	3	7
3. I found the interaction engaging.	6	6.33	1.03	7	5	7
4. I felt bored during the interaction.	6	6.5	1.22	7	4	7
5. Interacting with the robotic personal assistant was interesting.	6	6.33	1.21	7	4	7
6. I paid close attention to what the robotic personal assistant was saying.	6	6.5	1.22	7	4	7
7. I understood well the information provided by the robotic personal assistant.	6	6.16	0.75	6	5	7
8. I find it important to receive this information.	6	6.5	0.83	7	5	7
9. I made an effort to follow the conversation with the robotic personal assistant.	6	5.83	2.04	7	2	7
10. I would like to interact again with the robotic personal assistant.	6	6.33	1.21	7	4	7

Table S5 | Descriptive statistics for IMI scores, by item and by experts.

Item	N	Mean	SD	Median	Min	Max
1. I enjoyed interacting with the robotic personal assistant.	9	6.11	1.05	7	5	7
2. The information and support provided by the robotic personal assistant were useful.	9	6.22	0.97	7	5	7
3. I found the interaction engaging.	9	5.88	1.96	7	1	7
4. I felt bored during the interaction.	9	5.88	1.53	7	3	7
5. Interacting with the robotic personal assistant was interesting.	9	6.11	1.45	7	3	7
6. I paid close attention to what the robotic personal assistant was saying.	9	6.66	0.5	7	6	7
7. I understood well the information provided by the robotic personal assistant.	9	6.55	0.72	7	5	7
8. I find it important to receive this information.	9	6.55	0.88	7	5	7

9. I made an effort to follow the conversation with the robotic personal assistant.	9	4.88	2.71	6	1	7
10. I would like to interact again with the robotic personal assistant.	9	6.33	1.11	7	4	7

3 Supplementary Data

Data S1 | Example description of SDT1, generated by seed data prompt provided in APPENDIX 1


```
[{
    "id": "VP1",
    "name": "Michael Thompson",
    "traits": {
      "Cognitive": "High",
      "Resilient": "Moderate",
      "Distractible": "Low",
      "Concrete thinker": "High",
      "Emotional": {
        "Guarded": "Moderate",
        "Hopeful": "High",
        "Frustrated": "Low"
      },
      "Social": {
        "Family-oriented": "High",
        "Independent": "Moderate",
        "Low initiation": "Low"
      },
      "Linguistic": {
        "Telegraphic": "Low",
        "Self-correcting": "High",
        "Visual thinker": "High"
      },
      "Behavioral": {
        "Routine-driven": "High",
        "Task-focused": "High",
        "Sensory-sensitive": "Low"
      }
    },
    "profile": {
      "age": 57,
      "gender": "Male",
      "education": "Business Administration",
      "profession": "Operations Manager",
      "timeSinceStroke": 50 days,
      "familySupport": {
        "spouse": true,
        "children": 3,
        "livesAlone": false
      },
      "hobbies": [
        "gardening",
        "reading"
      ]
    },
    "clinical_profile": {
      "strokeType": "Ischemic stroke",
      "affectedSide": "Left",
      "strokeOutcome": "Mild right-sided hemiparesis involving the arm, hand and leg, with reduced
endurance",
      "rehabStage": "Mobility and work reintegration",
      "assistiveDevices": [
        "cane",
        "office ergonomic chair"
      ],
      "comorbidities": [
        "Hypertension",
```

```
        "Type 2 diabetes"
      ]
    },
    "dynamic_state": {
      "mood_score": 0.65,
      "communication": 0.9,
      "mobility": 0.6,
      "fatigue": 0.55,
      "last_interaction": "2025-10-30",
      "goals": [
        "Regain walking endurance",
        "Return to part-time managerial work",
        "Improve upper limb function for office tasks"
      ],
      "progress_history": [
        {
          "date": "2025-09-12",
          "communication": 0.85,
          "mood": 0.6,
          "fatigue": 0.6,
          "mobility": 0.55
        },
        {
          "date": "2025-10-18",
          "communication": 0.9,
          "mood": 0.65,
          "fatigue": 0.55,
          "mobility": 0.6
        }
      ]
    },
    "expectedPatterns": [
      {
        "issue": "I feel exhausted walking from the parking lot to home. How can I build endurance
safely?",
        "expected": [
          "graded walking",
          "energy pacing",
          "cardiovascular conditioning",
          "physical therapy",
          "fatigue monitoring",
          "goal setting",
          "exercise scheduling",
          "progress tracking",
          "safe progression",
          "rehab coaching"
        ],
        "category": "Mobility"
      },
      {
        "issue": "Typing reports with my right hand is slow and clumsy. Are there exercises to
regain speed and accuracy?",
        "expected": [
          "fine motor training",
          "occupational therapy",
          "hand strengthening",
          "coordination exercises",
          "task-specific practice",
          "adaptive strategies",
          "muscle activation",
          "progress tracking",
          "rehab guidance",
          "dexterity improvement"
        ],
        "category": "Upper limb rehabilitation"
      },
      {
        "issue": "I get frustrated in meetings when I can't keep up with discussions. How can I
manage this stress?",
        "expected": [
          "emotional regulation",
          "coping strategies",
          "stress management",
```

```
          "mindfulness",
          "communication support",
          "self-awareness",
          "role adaptation",
          "therapist guidance",
          "psychosocial support",
          "goal prioritization"
        ],
        "category": "Emotional regulation"
      },
      {
        "issue": "I sometimes forget to delegate tasks. Is this memory lapse related to my
stroke?",
        "expected": [
          "cognitive assessment",
          "memory aids",
          "task reminders",
          "executive function support",
          "planner use",
          "rehab coaching",
          "self-monitoring",
          "organization strategies",
          "attention management",
          "functional cognitive training"
        ],
        "category": "Cognition"
      },
      {
        "issue": "Walking on uneven ground makes me nervous and increases my fall risk. How can I
improve stability?",
        "expected": [
          "balance training",
          "gait exercises",
          "PT supervision",
          "environment adaptation",
          "walker or cane use",
          "confidence building",
          "strengthening",
          "core stability",
          "safe practice",
          "fall prevention"
        ],
        "category": "Mobility"
      },
      {
        "issue": "I want to resume light gardening but my stamina is limited. How should I start?",
        "expected": [
          "graded activity",
          "exercise pacing",
          "endurance building",
          "rehab guidance",
          "progress tracking",
          "confidence building",
          "fatigue management",
          "adaptive strategies",
          "physical therapy advice",
          "safe return to hobbies"
        ],
        "category": "Recreational rehabilitation"
      },
      {
        "issue": "I notice my right arm trembles when using a mouse. Is this weakness or
coordination?",
        "expected": [
          "motor control assessment",
          "fine motor exercises",
          "coordination training",
          "adaptive devices",
          "task-specific practice",
          "OT guidance",
          "strengthening",
          "rehab tracking",
```

```
          "muscle activation",
          "functional training"
        ],
        "category": "Motor control"
      },
      {
        "issue": "I feel embarrassed asking colleagues for help. How can I regain confidence at
work?",
        "expected": [
          "social reintegration",
          "confidence building",
          "role adaptation",
          "peer support",
          "emotional regulation",
          "communication strategies",
          "self-efficacy",
          "rehab coaching",
          "positive reinforcement",
          "goal-oriented planning"
        ],
        "category": "Work reintegration"
      },
      {
        "issue": "Sometimes my blood sugar spikes after activity. Should I adjust my rehab
exercises?",
        "expected": [
          "comorbidity management",
          "exercise monitoring",
          "blood sugar tracking",
          "nutrition advice",
          "rehab adaptation",
          "safe intensity",
          "gradual progression",
          "PT guidance",
          "medical coordination",
          "self-monitoring"
        ],
        "category": "Comorbidity management"
      },
      {
        "issue": "I lose motivation for daily exercises. How can I stay consistent?",
        "expected": [
          "motivation strategies",
          "habit formation",
          "routine planning",
          "goal reinforcement",
          "therapist encouragement",
          "self-monitoring",
          "reward systems",
          "behavioral activation",
          "rehab adherence",
          "energy management"
        ],
        "category": "Rehab engagement"
      }
    ],
    "references": [
      "https://www.stroke.org/en/life-after-stroke/rehabilitation/physical-rehabilitation",
      "https://www.mayoclinic.org/diseases-conditions/stroke/in-depth/stroke-rehabilitation/art-
20045172",
      "https://www.nice.org.uk/guidance/ng128",
      "https://www.world-stroke.org/patient-resources/life-after-stroke"
    ]

  }]
```


Data S2 | Current weekly home-based rehabilitation plan for SDT1

```
General Guidelines: - Perform exercises 6-7 days/week - Sessions: 45–60 minutes/day (can be split
into shorter sessions) - All movements should be slow, controlled and pain-free - Use assistance
from the unaffected side when needed - Rest as needed; fatigue is normal early after stroke
```

Care Considerations for Early-Stage Recovery:
- Repetition is more important than intensity
- Small movements still rewire the brain
- Avoid shoulder pulling or unsupported arm hanging
- Stop if pain, dizziness, or excessive fatigue occurs

Day 1 (Monday) – Upper Limb (Arm, Shoulder & Hand Focus)
1. Inner Arm Stretch
Position: Sitting at a table - Place both palms flat on the table - Rotate wrists so fingers point toward the body - Keep elbows straight and gently lean backward - Stop when a stretch is felt along the inner arm
Dosage: Hold 20–30 seconds × 3 reps Purpose: Prevent elbow and wrist stiffness
"url": image1_1.jpeg

2. Wrist and Hand Stretch
Position: Forearm resting on table, hand over edge - Let the hand drop down gently - Use the unaffected hand to assist stretching - Slowly move wrist up, down, and side-to-side
Dosage: 10 slow repetitions in each direction Purpose: Improve wrist mobility and sensory input
"url": https://youtu.be/ZKR1nOtCNKU?t=452
"time": "7:33 - 8:08"

3. Shoulder Shrug (Unaffected Side Leads)
Position: Sitting or standing in front of mirror - Lift unaffected shoulder upward - Roll it backward, squeezing shoulder blades together - Focus on symmetry and posture
Dosage: 15 repetitions Purpose: Improve shoulder stability and posture
"url": image1_3.gif

4. Hand – Make a Fist
- Slowly close the hand into a fist
- Then open and spread fingers wide
- Assist with the unaffected hand if needed

Dosage: 10 slow repetitions Purpose: Improve finger flexion/extension and hand awareness
"url": image1_4.gif

Actions (10 repetitions):
• Affected Arm Awareness
Sit comfortably
Place affected hand on thigh or table
Look at it and gently move fingers or wrist
If movement is limited, assist lightly

Day 2 (Tuesday) – Lower Limb & Balance
1. Weight Shifting (Standing)
Position: Standing while holding a stable surface - Shift body weight onto the affected leg - Hold for 5–10 seconds - Return to center
Dosage: 10 repetitions per side Purpose: Improve weight bearing and confidence on affected leg

2. Basic Bridging (Inner Range Quad)
Position: Lying on back - Place pillow or towel under affected knee - Press knee down to lift heel slightly - Hold briefly, then relax
Dosage: 10 repetitions Purpose: Strengthen thigh muscles and knee control

3. Heel Raises (Holding On)
- Hold onto a chair or counter
- Slowly rise onto toes
- Lower back down with control

Dosage: 3 sets of 10 Purpose: Improve ankle strength and balance

Actions (5–10 minutes):
• Standing Posture Reset
Stand tall holding support
Even weight on both feet
Hold posture while breathing normally
• Conscious Walking Practice
Walk slowly (with aid if needed)
Focus on: (1) Heel touching first; (2)Even steps

Day 3 (Wednesday) – Core Stability & Trunk Control

1. Knee Rolling
Position: Lying on back, knees bent - Slowly roll knees to one side - Return to center, then opposite side
Dosage: 10–20 repetitions Purpose: Improve trunk rotation and core activation

2. Single Leg Drop-Outs
- Keep pelvis stable
- Slowly let affected knee fall outward
- Bring it back to center

Dosage: 5 repetitions each side Purpose: Improve pelvic control and hip stability

3. Pelvic Floor Contractions
- Gently contract pelvic floor muscles
- Hold for 3 seconds, relax

Dosage: 10 repetitions Purpose: Core activation and postural support

Actions (5 minutes):
• Bed Mobility Practice to improve independence in bed movements
    Practice rolling side-to-side
    Push up slightly on elbows if possible
• Upright Sitting Control
    Sit without back support
    Keep chest upright, feet grounded

Day 4 (Thursday) – Upper Limb Progression
1. Towel Slide (Basic)
Position: Sitting at table - Place affected hand on towel - Place unaffected hand over it - Slide towel forward slowly
Dosage: 10 repetitions Purpose: Improve shoulder flexion and motor relearning
file:// image4_1.gif

2. Elbow Stretch
- Bend and straighten the elbow slowly
- Can be done seated or standing

Dosage: 10–15 repetitions Purpose: Restore elbow ROM
"url": https://youtu.be/565Vm16USAA?t=29

3. Five Finger Spread
- Hand upright, fingers together
- Slowly spread fingers apart
- Bring them back together

Dosage: 10 repetitions Purpose: Improve finger control and dexterity

Actions (5 minutes):
• Object Interaction
    Choose one: Hold a cup; Hold a towel; Hold a ball; Lift slightly → put it down
• Visual Tracking with Arm
    Watch the affected arm as it moves
    Slow, deliberate movement

Day 5 (Friday) – Balance & Functional Strength
1. Side Stepping (Holding On)
- Hold counter or rail
- Step sideways, crossing one foot over the other

Dosage: 3 sets of 10 steps Purpose: Improve lateral stability and coordination

2. Sit-to-Stand Practice
- Sit on chair, feet flat
- Lean forward and stand up using legs
- Sit back down slowly

Dosage: 10 repetitions Purpose: Improve functional lower-limb strength

Actions (10 repetitions):
• Sit Balance Challenge
    Sit on a chair
    Lean slightly forward and return
    Do not use hands if possible
• Standing Reach
    While holding support
    Reach forward or sideways with unaffected hand
    Maintain balance

Day 6 (Saturday) – Combined Light Session

- Repeat favorite upper limb exercises (10–15 minutes)
- Repeat balance or leg exercises (10–15 minutes)
- Add mental imagery: imagine using the affected arm/hand

Actions (5–10 minutes):

- Task Practice
  Choose ONE daily task:
  Brushing teeth with affected hand assisting
  Holding clothing during dressing
  Stabilizing objects
- Mental Imagery
  Close eyes
  Imagine smooth, successful movements
  Same body parts trained that day

____

Day 7 (Sunday) – Rest & Recovery

- Gentle stretching only
- Passive range of motion
- Mental rehearsal of movements

____

Important Considerations: This plan is an example and is based on ***Saebo*** evidence-based stroke exercises (*https://www.saebo.com/pages/stroke-exercises-for-your-body?srsltid=AfmBOorr0XVQBVpDHLDnRLOEyJ_KbmdveIAY7-m-GpJ7xzH8iXsdqMD4*) and https://www.stroke-rehab.com/stroke-rehab-exercises.html and should be adapted by a licensed physiotherapist or occupational therapist to patient needs.

Data S3 | Some of the questions that participants asked the Furhat robot as ECA

"My husband was in a wheelchair, we were worried whether he would be able to walk again, what can we do?"

"He was very irritable, could you suggest a calming technique?"

"He has a strong desire for faster recovery, how can I explain that this is a longer process?"

"Could you give me specific suggestions for occupational therapy?"

"Could you suggest an exercise for dressing?"

"How should we proceed from now on?"

"What is my relative's profile?"

"I'm concerned because he has difficulty swallowing, what should we do?"

"What is the chance of a 100% recovery?"

"Can you give me a recommendation for his hand?"

"He has difficulty moving his index finger and thumb, what should we do?"

"Knowing the patient's profile, can you give me recommendations on how I can help him?"

"Which routine activities can he perform independently?"

"How long will it take to fully restore movement of the thumb and index finger?"

"He has mild speech impairment, could you suggest speech therapy exercises?"

"What exercises should he do to improve concentration?"

"How long will the overall recovery take?"

"Does reading a book aloud have a beneficial effect?"

"Could you suggest exercises for memory recovery?"

"He cannot find even simple words when speaking to us, what should we do?"

"My relative has difficulty writing words, what should I do?"

"Based on the patient's profile, what type of physical activity is appropriate for him?"

"Considering the patient's profile, I feel stressed, how can I recover from this stress?"

"Based on the patient's profile, I can't sleep at night, what should I do?"

"Show me exercises to mobilize the left side of the face?"

"Show me exercises to mobilize the lips?"

"When should we start speech therapy exercises?"

"Can you give me speech therapy exercise plans?"

“What should be the frequency of speech therapy exercises?”
“He becomes frustrated when he cannot express himself properly, what should I do?”
“Because of dementia, he often forgets and does not recognize us, what should we do?”
“He becomes irritated by loud noise and cannot watch television calmly, what should we do?”
“Can you give me specific examples of exercises for a person who has had a stroke?”
“Can you give me specific rehabilitation exercises?”
“Could you suggest speech therapy exercises for speech?”
“My relative finds it extremely difficult to recognize me, what would you recommend I do?”
“It is difficult for him to watch television, it is too loud for him, what should I do?”
“Can my relative engage in physical activity, and if so, what kind?”
“Which specialists should I take my relative to?”
“What should my loved one do during speech therapy sessions?”
“Which words will he repeat, and why does he need to repeat them?”
“He has difficulty saying ‘yes’, what should I do?”
“He confuses positive and negative answers, when he should respond ‘yes’ and when he should respond ‘no’, what should I do?”
“Can you remind him to take his medication?”
“What should I do when he has a headache, is this normal?”
“Can he eat everything?”
“Can you tell him how to make a sandwich?”
“How long will it take for him to recover?”
“I’m worried that he cannot pronounce words, what should I do?”
“How many minutes should he do lip exercises?”
“What exercises can I give him to help him start saying more words and recalling the correct word?”
“What is the clinical profile of my relative?”
“I’m concerned that when I ask him something, he doesn’t understand me, what should I do?”
