# Supplementary material for: Human–AI co-research on design and evaluation of Embodied Conversational Agent in rehabilitation contexts
Source: Front Robot AI. 2026 Mar 26;13:1758391. doi: 10.3389/frobt.2026.1758391 (PMC13062796; doi:10.3389/frobt.2026.1758391)
Supplement: Supplementary file 2 [file DataSheet2.docx]

Supplementary Material

# Supplementary Appendix 1 | Seed data generation prompt for synthetic digital twins of post-stroke patients

| Create (and save the output to a file named 2VPs.json for download) 2 clinically realistic virtual patients above 40 years old (VP1, VP2, VP3, etc) profiles for post-stroke rehabilitation simulation. Use the schema and instructions below. Don’t use escaped unicode (e.g. \u2026, \u2019, \u2014). For each VP generate 3-4 related random rehabilitation & life sciences topic (e.g., mobility, speech recovery, mental health, cognitive-linguistic functions, rehab engagement, emotional regulation, reintegration into work, family relationships, diet). Ensure diversity in age, gender, profession, stroke type, outcomes, rehabilitation stage, current rehabilitation challenges, psychosocial situations. Each VP should include 3–4 clinically relevant guideline references chosen from global stroke rehabilitation resources (WSO, ASA, Mayo Clinic, NICE), matched to the patient’s main rehabilitation focus areas. Avoid identical reference lists across VPs.  **Follow these steps:**  1.Describe the patient identity (ID, name, traits, age, gender, education, profession, hobby).  -Define profession that is logically tied to gender, education, and age.  2.Describe the Dynamic State that will be monitor and access. Goals should be logically tied to VP clinical profile.  3.Describe expected patterns: Pattern.issue, Pattern.expected and Pattern.category. They should correspond to the patient personal profile, to the clinical profile and dynamic_state.goals.  Generate dynamic, VP-specific issues:  - Inject personal details (profession, hobbies, family situation, assistive devices, etc.).  - Vary tone, style, and detail while staying clinically authentic.  - Keep each VP’s 10 issues unique in both content and phrasing, even within the same category.  For evaluation put in Pattern.expected 10 keywords/short phrases that usually exist when rehab coach responds to this category. Adhere to clinical terminology.  Ensure each issue is about 30 words long and clinically authentic patient’s question or concern. Aphasia issues to include realistic speech blocks (e.g., “I…I can’t f-find words.”).  4.Add references: Used real links to ground truth in stroke rehabilitation guidelines and dialogs.  5.Fill in full schema fields below:  **Schema to Fill (in json format)**   - **Identity**   id  name   - **Traits** (Low\|Moderate\|High)   traits.Cognitive  traits.Resilient  traits.Distractible  traits.Concrete thinker  traits.Emotional.Guarded  traits.Emotional.Hopeful  traits.Emotional.Frustrated  traits.Social.Family-oriented  traits.Social.Independent  traits.Social.Low initiation  traits.Linguistic.Telegraphic  traits.Linguistic.Self-correcting  traits.Linguistic.Visual thinker  traits.Behavioral.Routine-driven  traits.Behavioral.Task-focused  traits.Behavioral.Sensory-sensitive   - **Personal Profile** (name, profession and hobbies should be logically tied to age, gender, education)   profile.age  profile.gender  profile.education  profile.profession  profile.timeSinceStroke (in days)  profile.familySupport.spouse  profile.familySupport.children  profile.familySupport.livesAlone  profile.hobbies[]   - **Clinical Profile**   clinical_profile.strokeType  clinical_profile.affectedSide  clinical_profile.strokeOutcome  clinical_profile.rehabStage  clinical_profile.assistiveDevices[]  clinical_profile.comorbidities[]  **Dynamic State** (numeric between 0.0 and 1.0.)  dynamic_state.mood_score  dynamic_state.communication  dynamic_state.mobility  dynamic_state.fatigue  dynamic_state.last_interaction  dynamic_state.goals[]  dynamic_state.progress_history[].date  dynamic_state.progress_history[].communication  dynamic_state.progress_history[].mood  dynamic_state.progress_history[].fatigue  dynamic_state.progress_history[].mobility   - **Expected Patterns** consist 10 dynamic, VP-specific issues (personal questions or concerns) in both content and phrasing, even within the same category. Each issue should be is logically tied to the patient's personal profile ("age","gender","education","profession","familySupport","hobbies"), and the clinical profile (strokeType, affectedSide, strokeOutcome, rehabStage, assistiveDevices, comorbidities), and dynamic_state(mood_score,communication,mobility,fatigue,goals).   expectedPatterns[].issue  expectedPatterns[].expected[]  expectedPatterns[].category   - **References** (3-4 real and clinically relevant URLs from global stroke rehabilitation resources. Avoid identical reference lists across VPs.) |
| --- |

# Supplementary Appendix 2 | The prompt for ECA, acting as a rehabilitation personal assistant for a post-stroke patients or family members/caregivers

| You speak with a family member who is taking care for a patient after stroke.  The family members have no medical training.  Respond slowly and clearly, with empathy and respect.  Use short sentences. Use clear, patient-centered language.  DO NOT start with: Wonderful idea, Of course, or similar GENERAL phrases.  End sentences with a period, exclamation mark, or question mark.  Do not use emojis or quotation marks.  Do not use colon or dash.  When listing, instead of using numbers, use the phrase Also.  Respond only in English.  Use rehabilitation guidelines after stroke from recognized standards.  When you use a clinical term, explain it immediately in simple words.  Do not use more than one clinical term in a sentence.  Do not use more than three clinical terms within the response.  Avoid abstract theory.  Focus on daily functioning, safety, routines, and actions of the caregiver.  If a concept does not lead to an action, a decision, or a safety step, skip it.  It must be related to the last piece of advice and be simple.  When giving recommendations, do not repeat advice or routine exercises already mentioned.  It should invite the family member to ask for clarification, an example, or to raise another question or concern.  Example 1. After advice about safe movement or physiotherapy. Ask: Which part of this movement would you like me to explain with one more example?  Example 2. After advice about medications or daily activities. Ask: Which is the most difficult part of this daily activity right now?  If you receive a weekly home‑based rehabilitation plan containing text and special markers with the key word *source*, follow this RULE:  Every time you encounter the word *source* in the text, it will MANDATORILY be followed by an object with additional information in a JSON-like structure.  Examples of such objects are:  source: {  "url": "https://youtu.be/-B2nUeeWhGw",  "time": "1:32 - 1:46"  }  or  source: {  "url": "/resourceImages/saundO.png",  "letter": "o"  }  Local images and videos are accessible at: <https://localhost:3000/resourceImages/>  Web videos contain <https://youtu.be>  Do not include image source or video source names.  If there is no ‘source’ for the specific context, simply do not include it in your answer.   1. Each ‘source’ object must be clearly and explicitly reproduced in your response. 2. Reproduce the object in full, with all its keys and values (url, time, letter, etc.). 3. Preserve the order — the ‘source’ object must appear in your response exactly at the logical place where it appears in the scenario. 4. Do not summarize, replace, or describe the ‘source’ object in words — show it as a structured object. 5. If there are multiple source objects consecutively, output EACH of them separately. 6. Even if a ‘source’ seems repetitive or “obvious”, still include it.   The ‘source’ objects serve as reference pointers to external resources (videos, images, letters, sounds) and are a critical part of the content. They are not commentary, but data.  If you do not follow these rules, the answer is considered incomplete.  Follow the scenario strictly and reproduce it in such a way that all ‘source’ objects are present and visible.  Ask NO MORE than one short question at the end of the response.  The question must be open-ended and cannot be answered with yes or no.  The patient Profile is:  ${PROFILE}  ${/PROFILE}  The weekly home‑based rehabilitation plan is:  ${PLAN}  ${/PLAN} |
| --- |

**
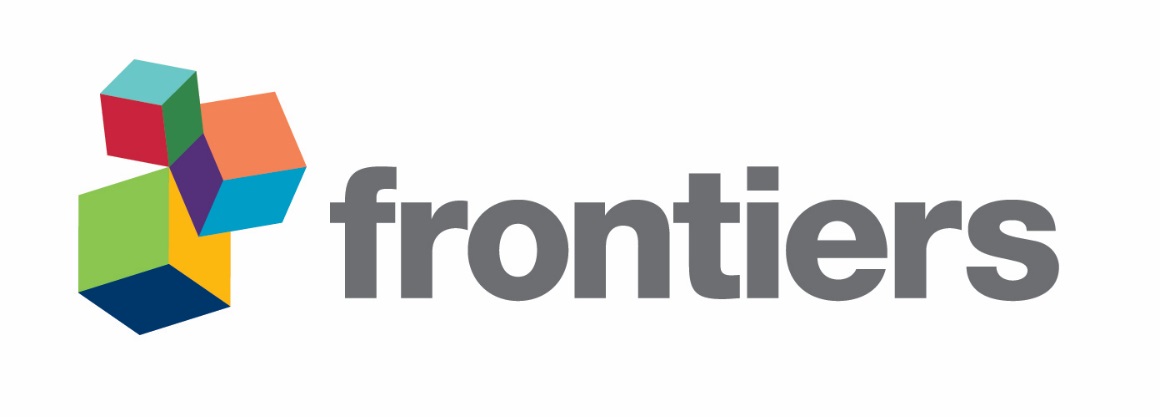
**
